# Mapping Interictal activity in epilepsy using a hidden Markov model: A magnetoencephalography study

**DOI:** 10.1002/hbm.26118

**Published:** 2022-10-19

**Authors:** Zelekha A. Seedat, Lukas Rier, Lauren E. Gascoyne, Harry Cook, Mark W. Woolrich, Andrew J. Quinn, Timothy P. L. Roberts, Paul L. Furlong, Caren Armstrong, Kelly St. Pier, Karen J. Mullinger, Eric D. Marsh, Matthew J. Brookes, William Gaetz

**Affiliations:** ^1^ Sir Peter Mansfield Imaging Centre, School of Physics and Astronomy University of Nottingham Nottingham UK; ^2^ Young Epilepsy St Pier's Lane Lingfield RH7 6PW UK; ^3^ Oxford Centre for Human Brain Activity University Department of Psychiatry, Warneford Hospital Oxford UK; ^4^ Department of Radiology Children's Hospital of Philadelphia Philadelphia Pennsylvania USA; ^5^ Aston Brain Centre Aston University Birmingham UK; ^6^ Pediatric Epilepsy Program, Division of Child Neurology CHOP Philadelphia Pennsylvania USA; ^7^ Centre for Human Brain Health, School of Psychology University of Birmingham Birmingham UK; ^8^ Departments of Neurology and Paediatrics University of Pennsylvania Perelman School of Medicine Philadelphia Pennsylvania USA

**Keywords:** epilepsy, hidden Markov model, interictal activity, magnetoencephalography

## Abstract

Epilepsy is a highly heterogeneous neurological disorder with variable etiology, manifestation, and response to treatment. It is imperative that new models of epileptiform brain activity account for this variability, to identify individual needs and allow clinicians to curate personalized care. Here, we use a hidden Markov model (HMM) to create a unique statistical model of interictal brain activity for 10 pediatric patients. We use magnetoencephalography (MEG) data acquired as part of standard clinical care for patients at the Children's Hospital of Philadelphia. These data are routinely analyzed using excess kurtosis mapping (EKM); however, as cases become more complex (extreme multifocal and/or polymorphic activity), they become harder to interpret with EKM. We assessed the performance of the HMM against EKM for three patient groups, with increasingly complicated presentation. The difference in localization of epileptogenic foci for the two methods was 7 ± 2 mm (mean ± SD over all 10 patients); and 94% ± 13% of EKM temporal markers were matched by an HMM state visit. The HMM localizes epileptogenic areas (in agreement with EKM) and provides additional information about the relationship between those areas. A key advantage over current methods is that the HMM is a data‐driven model, so the output is tuned to each individual. Finally, the model output is intuitive, allowing a user (clinician) to review the result and manually select the HMM epileptiform state, offering multiple advantages over previous methods and allowing for broader implementation of MEG epileptiform analysis in surgical decision‐making for patients with intractable epilepsy.

## Graphical abstract

In this study, we use hidden Markov modeling (HMM) to create a unique statistical model of interictal brain activity for 10 pediatric epilepsy patients. This data‐driven model produces an output unique to each patient, and is used to localize the epileptogenic area(s). In two patients, where more than one focus is identified, the HMM provides additional information about the relationship between the epileptiform activity arising in those areas.

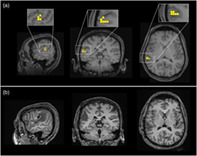

## 1 INTRODUCTION

Epilepsy is a neurological disorder affecting ~50 million people worldwide (Beghi et al., 2019). It is characterized clinically by the occurrence of seizures which are generated by abnormal electrical cellular discharges in the brain. Epilepsy falls broadly into two categories: focal or generalized: in patients with focal epilepsy, there can be a single focus or multiple discreet foci that generate seizures, whereas for generalized epilepsy, seizures originate from diffuse areas throughout the cortex and/or deep brain structures such as the thalamus or hypothalamus. For many patients, antiepileptic medications can control both the severity and frequency of seizures. However, ~30% of patients do not respond completely to medications (Mohan et al., 2018) and for these patients, surgical resection of affected brain tissue could be a viable treatment. However, this necessitates extensive presurgical planning to accurately locate the affected brain area(s) prior to resection.

Current evaluation of pharmaco‐resistant epilepsy is accomplished using electroencephalography (EEG; which measures electrical activity in the brain via assessment of electrical potentials at the scalp) alongside clinical factors and structural assessment using magnetic resonance imaging (MRI). This may be augmented by the nuclear medicine techniques of positron emission tomography and especially ictal (during seizure) single photon emission computed tomography. If candidate epileptogenic locations are identified via imaging, a more invasive intracranial EEG (iEEG) may be initiated where electrodes are located on the brain surface or within the grey matter. This allows electrophysiological assessment with optimal sensitivity and spatial accuracy, prior to resective surgery. Despite this extensive surgical planning, fewer than 50% of patients are seizure free 5 years postsurgery, with this number dropping to 38% at 10 years postsurgery (Mohan et al., 2018). It is therefore clear that a greater understanding of this heterogeneous disease, as well as improvements in clinical evaluation are required to improve patient outcome.

Magnetoencephalography (MEG) measures the magnetic fields induced by neuronal current flow. Unlike the electric potentials measured by EEG, magnetic fields are relatively unaffected by the high resistivity of the skull, resulting in less spatial distortion of the MEG compared with the EEG signal, and thus improved resolution. MEG is used in a growing number of clinical settings, particularly in epilepsy. Not only does MEG provide additional information about the location of the epileptogenic zone (Agirre‐Arrizubieta et al., 2014; Gofshteyn et al., 2019; Murakami et al., 2016; Nissen et al., 2016; Stefan et al., 2011), it can also be used to distinguish epileptogenic regions from eloquent cortex (Kim et al., 2013), and could be useful in mapping nonlesional or MRI negative focal epilepsies where there is no clear structural abnormality. Most importantly, a recent study by Rampp et al., (2019) showed, in 1000 patients, that presurgical MEG increases the chances of a patient achieving seizure freedom postsurgery when MEG localizations are resected.

MEG therefore has significant promise for assessment of patients with epilepsy, and the recent introduction of new technologies to capture the neuromagnetic field offer even higher spatial resolution, better sensitivity, and improved practicality, at lower cost (Boto et al., 2018, 2021; Brookes et al., 2022; Hill et al., 2020). This means that MEG could become even more established as the technique of choice for epilepsy evaluation. However, the detection of epileptogenic activity in MEG data remains a significant challenge. Recording data during a seizure is difficult due to uncontrolled patient movement, and for this reason, most MEG recordings are limited to interictal (between seizure) assessment (although see e.g., Tang et al., 2003). Interictal events—sharply contoured atypical signals (known as epileptiform activity, i.e., spikes, sharps, etc.) are observable in resting MEG (and EEG) data and are generally assumed to originate from seizure onset zones, meaning that spatially mapping their origin offers useful information on the location of epileptogenic cortex. However, detecting interictal discharges can be challenging for two reasons. First, they are sporadic and unpredictable and, in some patients, rare. Thus, capturing interictal epileptiform activity can sometimes be a challenge without lengthy recording sessions (which should ideally include natural sleep because interictal discharges are often enhanced or only present in sleep). Even when they do occur, it can take significant time for a neurophysiologist to identify, mark, and categorize them (manually) in a MEG recording due to the high channel density relative to clinical EEG (i.e., >250 channels compared with <30).

Second, the temporal morphology of epileptogenic activity can vary markedly between patients; some produce spikes with/or without slow wave activity, which varies dramatically in amplitude. Other patients generate polymorphic bursts of “sharp wave” activity characterized by high temporal frequency signatures. In some individuals, the pattern of epileptiform activity repeats (like a template), in others it differs on each occurrence. This makes automatic detection algorithms challenging to design.

There are two commonly used analysis methods for localization of interictal activity, equivalent current dipole (ECD) fitting and excess kurtosis mapping (EKM). In ECD, interictal spikes are inspected visually (Bagić et al., 2011). Once identified at the sensor level, a current dipole model is used to approximate the measured magnetic field just prior to, or at the peak of, the spike; by letting the origin of the modeled dipole vary spatially, and then selecting the point at which the model best fits the measured field, it becomes possible to localize the brain region generating the spike (Ebersole, 1997; Wheless et al., 1999). This technique works reasonably well in cases where high amplitude spikes are observed in isolation but is often not useful in cases where interictal activity includes polymorphic bursts. In addition, multifocal epilepsies are a challenge since ECD requires a priori estimation of the number of active regions.

In contrast, EKM (Gaetz et al., 2015; Robinson et al., 2004; Schwartz et al., 2010) is an automatic method which assumes only that the epileptiform signals of interest are sharply contoured (relative to the typically rhythmic background MEG signals). Kurtosis is a measure of the shape of a statistical distribution; in cases of abnormal activity (e.g., if a dataset has large spikes), it's statistical distribution includes a large “tail” and thus begins to look non‐Gaussian; this is quantified by increased kurtosis (also known as the fourth moment of the distribution). By application of a kurtosis algorithm to MEG signals extracted from multiple brain locations, it becomes possible to localize areas generating abnormal activity. EKM does not require a priori estimation of the number of epileptogenic regions; further, it is not limited to spikes, but can be used to assess any atypical activity, provided it has high kurtosis. However, EKM also has limitations; it has low sensitivity to low‐amplitude polymorphic activity. Also, counterintuitively, in cases with rapidly occurring high‐amplitude spikes, excess kurtosis has diminished sensitivity, because the kurtotic signals are so common they begin to represent the mean. For these reasons, neither ECD nor EKM is a perfect solution to analysis of MEG data in epilepsy, and other methods, which can accurately and automatically identify epileptiform activity, map its spatial origins, and (in multifocal epilepsy) characterize relationships between regions, would be useful.

Hidden Markov modeling (HMM) has gained traction in recent years as a method to elucidate complex neural dynamics in MEG data (Higgins et al., 2021; Quinn et al., 2018; Vidaurre et al., 2018). The method works by detection of repeated patterns of activity (known as states) in MEG data; patterns can be characterized based on a number of features, including amplitude, channel covariance, and spectral properties. In this article, we aimed to test the hypothesis that “an HMM could be used to identify, in time and space, epileptiform activity in agreement with current state of the art methods”. We further aimed to show that, “in at least some patients, our method could offer more information than the established EKM technique”. In what follows, we describe our data processing pipeline, and its application to MEG data acquired in multiple different patient groups, ranging from “simple” cases with focal spike‐and‐wave epileptiform activity for a single locus, to more complex cases with multifocal epileptiform activity exhibiting polymorphic bursts from multiple loci.

## 2 METHODS

### 2.1 Patient identification and data collection

This study was determined by the IRB to have exempt ethics status as it constituted secondary analysis of data captured for clinical purposes under the NIH common rule (January 2019). All research subjects were scanned as part of clinical care at the Children's Hospital of Philadelphia. Data from 10 pediatric patients undergoing presurgical epilepsy evaluation between the ages of 11 months and 17 years (median 8.18 years, see Table 1), were utilized in the study. There were two female and eight male patients. In total, 9 of the 10 had focal epilepsy with or without impaired awareness, and one (Patient 6) had epilepsy with combined focal and generalized features. Six patients had right focal epileptiform discharges, whereas 4 had left focal epileptiform discharges, as identified by clinical features and multimodal imaging methods including MEG analyzed with EKM. Most focal discharges were in the frontal or temporal regions with one patient (Patient 8) with central localization and one patient with both frontal and posterior temporal discharges (Patient 6). When known, etiology was primarily confirmed or suspected structural abnormality (such as focal cortical dysplasia), with one patient (Patient 1) with confirmed genetic etiology and two patients with, as yet unknown etiology.

**TABLE 1:** Patient characteristics.

Patient	Sex	Age at time of recordings	Etiology	Epilepsy type	Interictal discharge morphology	Location of EEG discharges	MEG category	Anatomical MEG location(s) and closest 10–20 electrode		Anesthesia used during MEG
								MEG site 1	MEG site 2 (if present)	
1	Male	6.73	Genetic	Focal with impaired awareness	Sharp waves	Right centrotemporal (C4/T4)	Focal spike wave	Right frontal, right precentral (C4)	N/A	General, propofol + dexmedetomidine + ondansetron
2	Male	9.62	Structural	Focal with preserved awareness	Sharp waves	Right frontal (F4/F8/F10)	Focal spike wave	Right anterior frontal (Fp2)	N/A	None
3	Male	5.67	Unknown	Focal with impaired awareness	Sharp waves	Left temporal (T3 at times shifted to F3)	Focal spike wave	Anterolateral left parietal C3)	N/A	General, propofol + dexmedetomidine + ondansetron
4	Female	4.93	Suspected structural	Focal with impaired awareness and secondary generalization	Sharp waves, spike waves and focal slowing	Right frontotemporal (F8 and T4/T6)	Focal spike wave	Right posterior frontal (**C4**/P4/T4)	N/A	General, propofol + dexmedetomidine + ondansetron
5	Female	17.04	Structural	Focal with preserved awareness	Sharp waves and spikes	Right frontotemporal (T4/T6, F8, T10)	Focal polymorphic	Right superior temporal gyrus, right middle temporal gyrus, right insula (**T4**/T6)	N/A	None
6	Male	11.55	Suspected structural	Combined	Sharp waves, focal slowing, and irregularly generalized discharges	Left frontal (F3 sharp waves and slowing), left posterior quadrant (T5/O1)	Focal polymorphic	Left anterior frontal and left Sylvian fissure (**F3**/F7)	N/A	General, propofol + dexmedetomidine + ondansetron
7	Male	16.52	Unknown	Focal with impaired awareness	Sharp waves	Right frontotemporal (F4/Fp2/F8)	Multifocal polymorphic	Right anterior and mid temporal (**T4**/F8)	F7	None
8	Male	0.96	Structural	Focal with preserved awareness	Sharp waves	Right central (C4/Cz)	Multifocal polymorphic	Right perirolandic, right anteromedial occipital lobe and hippocampal tail (C4/Cz/T4)	N/A	General, dexmedetomidine
9	Male	6.59	Structural	Focal with preserved awareness	Sharp waves	Left frontotemporal (T3/C3, F7/T3, and F3/C3)	Multifocal polymorphic	Left frontotemporal (**F7**/T3)	P3	General, propofol + dexmedetomidine + ondansetron
10	Male	11.88	Structural	Focal with preserved awareness	Sharp waves and focal slowing	Left frontotemporal (T3 slowing, T3/T5, Fp1/F3/F7)	Multifocal polymorphic	Left posterior temporal and left anterior to mid frontal (Fz/Cz/F3/F7/T5/T3)	N/A	General, propofol + dexmedetomidine +ondansetron

Bold typeface indicates the single most likely electrode.

Abbreviations: EEG, electroencephalography; MEG, magnetoencephalography.

Multiple 2‐min recordings were acquired using a CTF 275‐channel MEG system operating in third‐order synthetic gradiometer configuration. By collecting data in relatively short 2‐min runs, the maximum head motion within each dataset was minimized. The number of acquired datasets per patient ranged from 15 to 29; mean = 17.8. Data were acquired at a sample rate of 1200 Hz. In all cases, patients were scanned supine. Of the 10 patients, 7 were scanned whilst sedated using general anesthesia.

Prior to data acquisition, three head position indicator coils were attached to fiducial points on the head. During recording, these coils were energized (at nonphysiological AC frequencies) to allow continuous localization of their position relative to the MEG sensor array. All MEG scans were followed by an anatomical MRI during which MRI contrast markers were placed at the same fiducial points on the head. Coregistration of the MEG and MRI fiducial locations thus enabled complete spatial mapping of the MEG array relative to individual brain anatomy. This coregistration, in turn, allowed generation of functional images showing the cortical origins of epileptiform activity.

Given that the purpose of this work is to act as a feasibility study to test whether the HMM could be a useful tool in the identification and localization of epileptiform activity, patients were selected retrospectively to fall into three groups of increasing localization difficulty (see Table 1); four had focal epilepsy generating interictal spike and wave activity; two had focal epilepsy with only polymorphic bursts, and the final four had multifocal epilepsy with polymorphic bursts. These categories were determined by visual inspection of the MEG data (using EKM). Patient inclusion was determined first by CHOP Neurologists (Eric D. Marsh and Caren Armstrong), and visual inspection of all EKM findings was done by Eric D. Marsh, Caren Armstrong along with the technical assistance of MEG Scientist William Gaetz.

To assess the efficacy of our HMM MEG method, results from iEEG and/or surgical outcomes are provided for patients where these data exist (Patients 2, 5, and 8). A comparison with scalp EEG is also given in Table 1: The concordance of scalp EEG with EKM MEG results is given for each patient by comparing anatomical MEG localizations and their nearest 10–20 electrodes (bold typeface indicates the single most likely electrode), with the electrode positions of observed EEG discharges. Where our HMM MEG method was subsequently found to be concordant with EKM, it would therefore also be concordant with scalp EEG.

### 2.2 Data processing

**FIGURE 1:**
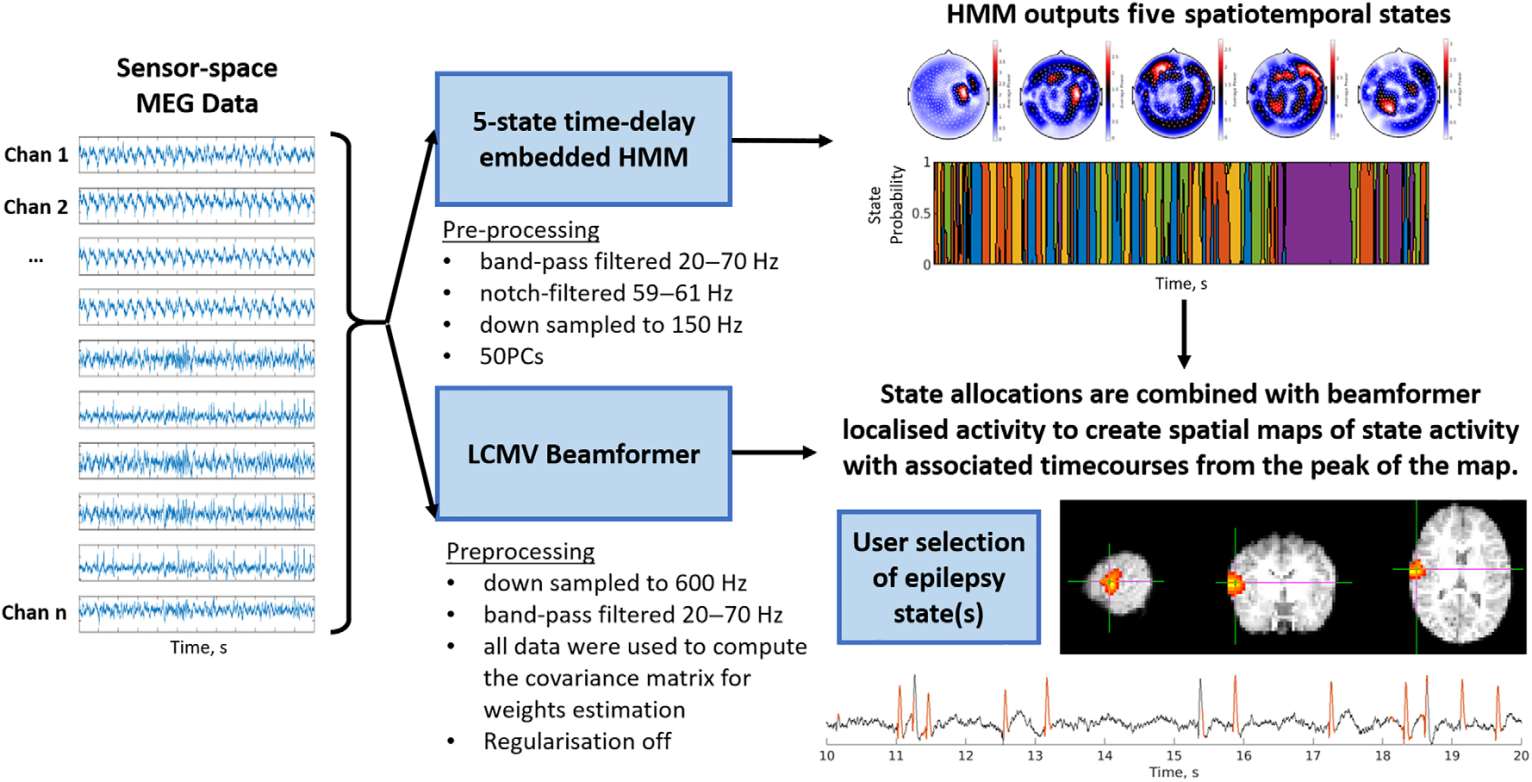
Schematic representation of the hidden Markov model (HMM)‐based process to identify epileptogenic activity. A multivariate time delay embedded HMM was used to identify five states, each state characterized by its mean, covariance (across channels), and spectral content. A beamformer was used, along with temporal state allocations, to generate images of state activity across the cortex and a time course of activity from the peak voxel. This allowed us to identify an epileptiform state and a map of epileptogenic cortex. LCMV, linearly constrained minimum variance; MEG, magnetoencephalography

Following MEG collection, data were visually inspected and any 2‐min runs containing obvious interference or segments where patient motion exceeded 1 cm were removed from further analysis. Since some patients have infrequent interictal activity, all data were checked by a MEG expert (William Gaetz) to ensure they contained epileptiform activity and runs which did not were subsequently discarded. This left an average of 11 runs per subject. Our HMM‐based method of mapping epileptiform activity comprised a two‐step process (see Figure 1): In step one, the HMM was applied to channel‐level MEG data to identify time periods in which interictal epileptiform activity occurred. In step two, a beamformer was used to localize the brain regions generating the activity identified by the HMM. These two steps are described in detail below.

#### 2.2.1 Hidden Markov modeling

To find spatiotemporal patterns corresponding to epileptogenic activity, we applied a multivariate, five‐state, time delay embedded HMM, in channel space. An HMM assumes that a series of recurring mutually exclusive “hidden” states govern the MEG data, such that every point in time is associated with one of the states. The sequence is assumed to be Markovian (i.e., the state active at a time point, *t*, only depends on that active at time point *t* − 1). An observation model links the HMM state to the observed values in the MEG data.

The HMM has been described extensively in previous papers (Baker et al., 2014; Vidaurre et al., 2018) and a complete mathematical description will not be repeated here. Briefly, in its simplest form, an HMM would describe each state using a multivariate distribution; that is, a mean (for all channels) and covariance (across channels). The five distributions that best described the data would be derived, and the probability of each data‐point belonging to a specific state would be calculated. The number of states is defined a priori and model inference would learn the sequence of states, from the observed data.

Here, we employed a more complex model which also allowed time‐delay embedding (Vidaurre et al., 2018), adding information on autocovariance (defined over a specified time window [duration 73 ms]). These state autocovariance patterns contain the spectral content of the signal, consequentially using our HMM, a single state is defined based upon signal variance, covariance across channels, and spectral content. This model had the potential to characterize both spike and wave activity and polymorphic bursts; in the former case the (typically high) amplitude of a spike, with a full width at half maximum of ~70 ms, coupled with its distinct spectral content would characterize the state and differentiate it from ongoing “normal” activity. In the latter case, since polymorphic bursts are associated with “sharps” (high‐frequency activity) we again reasoned that distinct spectral content would define the state.

Prior to application of the HMM, the data used for the model inference (comprising 266‐channel sensor space MEG data) were bandpass filtered between 20 and 70 Hz (to match the standard EKM pipeline used by the Children's Hospital of Philadelphia—see also below), notch filtered at 60 Hz (to remove mains frequency artifacts), temporally down sampled to 150 Hz, time‐embedded using 73 ms lags and a principal component analysis was used to reduce the data to 50 components (this allowed for faster model inference and helped to avoid overfitting). A total of fifty principal components were enough to capture 87% ± 3% of the data variance (mean and SD over subjects and runs). Note that each 2‐min clinical run was considered separately, just as the EKM data were. The model inference itself was undertaken using a variational Bayesian method which seeks to minimize the free energy of the system. We computed five‐states; for each state, in addition to an observation model, we obtained a time course of the probability that the state is active. These time courses were thresholded at two thirds, so the state was defined as “active” when the probability exceeded two thirds (Seedat et al., 2020). From the time courses, we also obtained a state‐transition‐matrix—a 5 × 5 matrix of probabilities defining the temporal relationship between states (i.e., element 2,1 in the matrix would represent the probability that State 1 followed State 2). This approach might offer useful information in cases with multifocal epileptiform activity where one source consistently precedes the other.

To assess the impact of the choice of model parameters used for the HMM inference, the model inference was computed multiple times for various lag durations (ranging from 33 to 207 ms) and for various numbers of states (ranging from 3 to 9). Please see the Supplementary Information S1 for further details.

#### 2.2.2 Beamforming

Following application of the HMM (in sensor space), a linearly constrained minimum variance beamformer (Robinson and Vrba, 1999) was used to localize the spatial signature of each state in the brain. The brain was divided into a regular 4 mm grid of voxels, and each voxel time course was defined as a weighted sum of sensor measures. The beamformer weights were defined using a data covariance matrix calculated in the 20–70 Hz frequency band, and a time window spanning the entire recording. To maximize spatial resolution, no regularization was applied (Brookes et al 2008). The forward field was calculated using a current dipole approximation and a multiple local sphere volume conductor model. The beamforming parameters (frequency filters and time windows for covariance estimation and regularization, as well as the choice of forward model) were selected to match the EKM method.

This resulted in a time course estimate of electrophysiological activity for each voxel location. To generate a functional map showing the spatial signature of each state, binary state time courses were imposed on voxel time courses to determine when each state was active and inactive. We then calculated the ratio of the variance when the state was active to the variance when the state was inactive, for every voxel time course in the brain, to highlight the brain regions which elicit changes in variance when the state switches on. This produced a spatial map of state activity. In addition to the spatial maps, we also used the beamformer to derive a time course of electrophysiological activity at the peak location of the spatial map. Beamformer weights were defined as above but using covariance calculated in the 1–150 Hz band, and a single time course showing 1–150 Hz activity was extracted.

Having derived a spatial map and time course of activity for each of the five states, these were visually inspected by a single MEG‐epilepsy expert (William Gaetz). Those states whose time courses showed epileptiform activity when the state was active were identified, and the spatial localization was noted for each run. These were termed the “epileptiform state(s).”

#### 2.2.3 Comparison to existing methods

We compared the results of our HMM, to the more established EKM technique. We selected EKM for this comparison because of its advantages over ECD (Hall et al., 2018) and its use in large pediatric cohorts (Gofshteyn et al., 2019). To ensure that a standard EKM pipeline was followed, we used commercial software developed by CTF (Coquitlam, BC, Canada) known as SAM(g2), and the established pipeline used clinically by the epilepsy team at the Children's Hospital of Philadelphia (Schwartz et al., 2010).

Prior to the application of EKM, all data were filtered 20–70 Hz. In the SAM(g2) implementation, the brain was divided into a regular grid of 5 mm voxels and a scalar beamformer (equivalent to that described above) was used to reconstruct electrophysiological signals at each voxel. A kurtosis value was then computed for the time course at every voxel, and voxels with a kurtosis value > 0.5 were marked as peaks (Gofshteyn et al., 2019; Schwartz et al., 2010). Having found the spatial locations of interest, 1–150 Hz voxel time courses were analyzed and temporal markers were placed at any point where the peak‐to‐RMS ratio exceeded 6 (Gofshteyn et al., 2019). This automated algorithm provided an estimate of both the regions and the time points which were likely generators of epileptiform activity. Following this, data were inspected visually by a single expert experienced in identification of epileptiform activity, to determine whether the abnormalities found by the algorithm were genuinely related to epilepsy or were generated by sources of no interest with high kurtosis (e.g., ocular or muscle artifact). Temporal markers unrelated to epileptiform activity were disregarded.

Following EKM, two separate measures were derived to quantitatively compare the output of the HMM and EKM mapping:

1. Spatial correspondence: Peak locations identified by the HMM (i.e., those regions whose variance increased when the HMM‐derived epileptiform state was entered) were compared with peak locations in kurtosis. We measured Euclidean distance between these peaks.
2. Temporal coincidence: We took all of the time points identified by our EKM pipeline as containing epileptic activity and determined the number which were temporally coincident with an occurrence of the epileptiform state. Here, temporal coincidence was defined as within 73 ms of the EKM marker (i.e., the approximate duration of a single spike, covering 11 points). Note that, the addition of a small amount of time around the EKM‐derived marker can mean that a single EKM marker is matched by more than one occurrence of the HMM epileptiform state; consequently, counterintuitively, values can exceed 100%.

All of the above (HMM, EKM, and their comparison) were applied to each 2‐min run, in each subject, separately. This meant 123 runs in total over 10 subjects.

The code used for model inference, computation of state‐wise functional maps, and comparison of the HMM method with EKM is available at https://github.com/ZSeedat/HMM_epilepsy.

## 3 RESULTS

Case study results for each of the three patient groups are shown here. For detailed analysis of all patient rs, please see the Supplementary Information S1.

### 3.1 Case 1: Focal spike and wave

**FIGURE 2:**
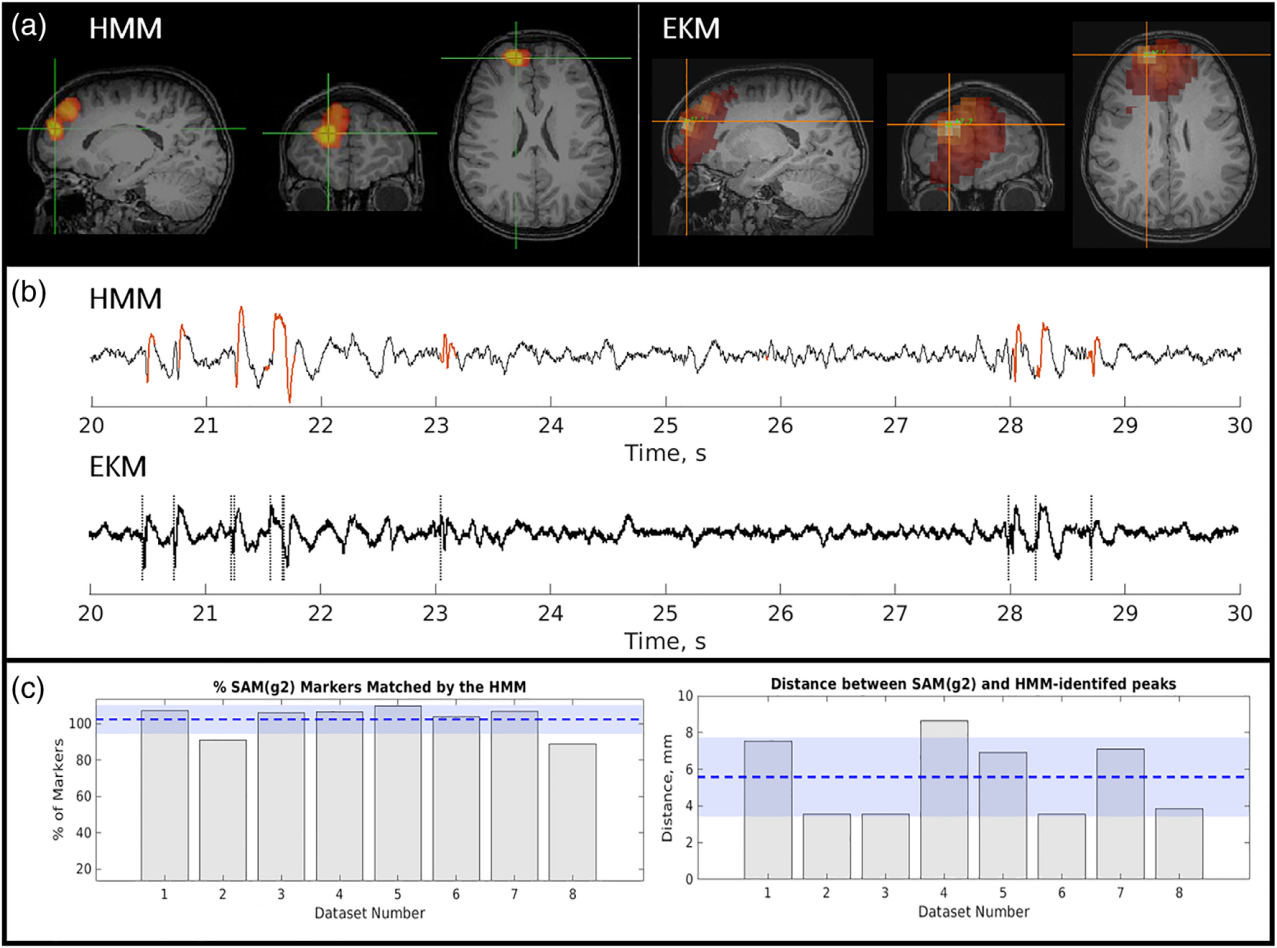
Epilepsy Case 1—Patient 2; focal, spike and wave. (a) The left‐hand side shows the spatial signature of the epileptiform state as defined by the hidden Markov model (HMM). The right‐hand side shows the spatial profile of excess kurtosis mapping (EKM). Both maps were thresholded for visualization. (b) Upper plot shows a beamformer‐derived time course from the peak location identified by the HMM with red regions showing occurrences of the HMM‐derived epileptiform state. The lower plot shows the equivalent time course from the peak in kurtosis. Dashed lines in the lower plot show time points of epileptogenic activity identified by EKM. (c) Quantitative analysis over eight runs from this subject. Left‐hand side shows temporal coincidence of EKM identified markers with the epileptiform state. Right‐hand side shows spatial correspondence. Dashed line and blue shading show the mean and SD of the measure over all eight 2‐min runs in this subject

Results for a single representative focal epilepsy patient are shown in Figure 2. Figure 2a shows the spatial signature of epileptogenic cortex, identified by the HMM (left) and EKM (right). These data are taken from a single run. Figure 2b shows example time course segments taken from the peak voxel in the HMM map (upper trace) and the EKM map (lower trace) (note the similarity between traces due to the close spatial correspondence of the peak source locations identified by each method). In the upper (HMM) trace, the time points at which the epileptiform state was active are shown in red. In the lower (EKM) trace, the timepoints identified as containing epileptiform activity are shown by the dotted line. Note the close temporal correspondence (at least for this small segment of data). These results are quantified in Figure 2c; the left‐hand bar chart shows the percentage of manually verified EKM markers that fell within the occurrence of the epileptiform state (i.e., our temporal coincidence metric). The right‐hand bar chart shows the Euclidean distance between the peak from the HMM, and the peak from the EKM. In both cases, the separate bars represent different 2‐min runs in the same subject.

The patient exhibited abundant, large amplitude spikes, with some slow wave activity. Spatial correspondence between EKM and HMM was 6 ± 2 mm (mean and standard deviation over eight 2‐min runs) and 102 ± 8% of EKM‐identified epileptic events were matched in time by the occurrence of the epileptiform state. Note that more than one state visit to a single kurtosis marker will occasionally yield values >100%. Across all datasets, the HMM epileptiform state was active for 4% ± 2% of the time.

**FIGURE 3:**
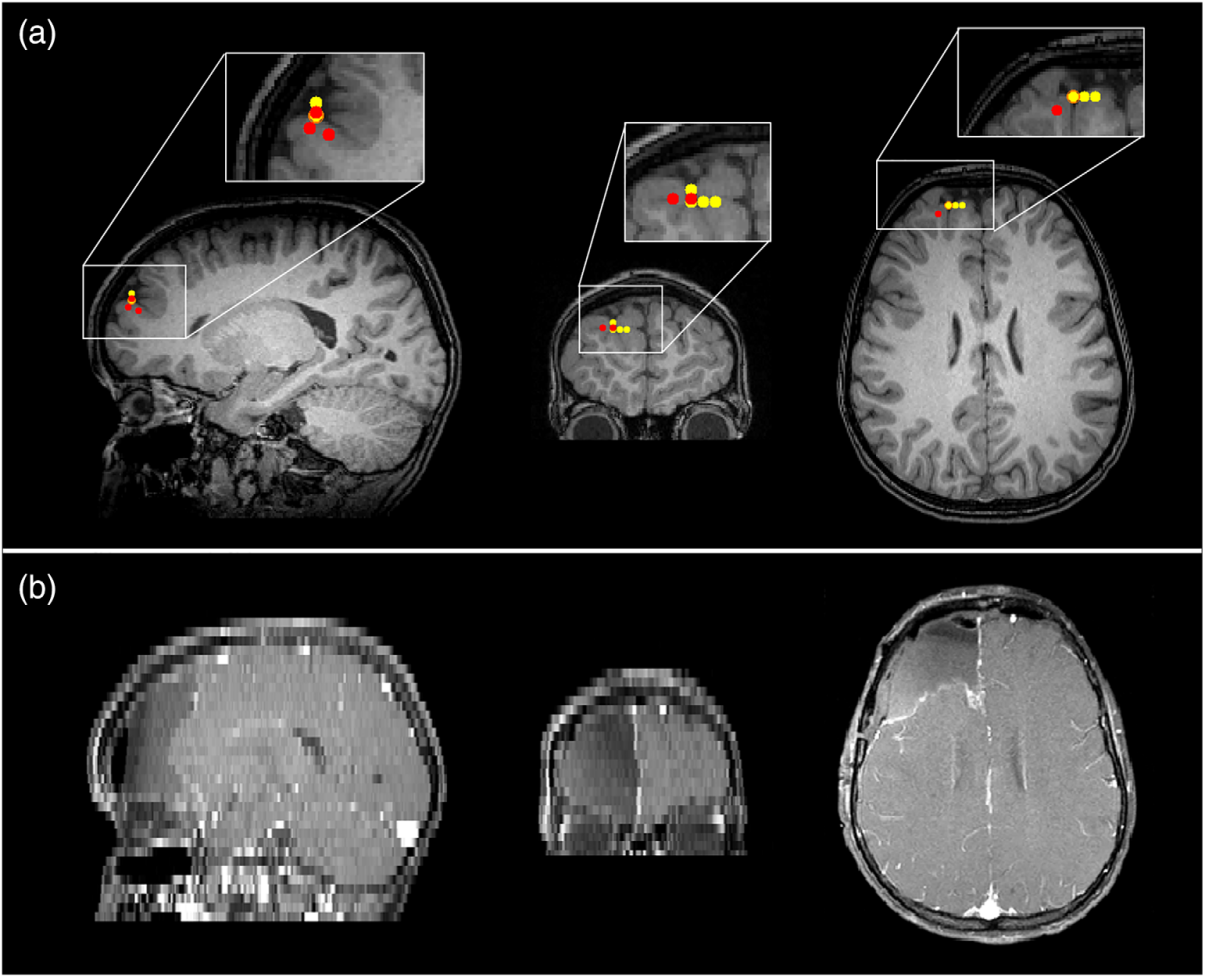
Epilepsy Case 1—magnetoencephalography (MEG) localizations compared with resected area for patient 2 (focal spike wave). Panel (a) The MEG localizations for the excess kurtosis mapping method (red markers) and the hidden Markov model method (yellow markers). Panel (b) shows the resected area in the postoperative magnetic resonance imaging (MRI). Note that the postoperative MRI has different resolutions in the sagittal, coronal, and axial planes

iEEG and postsurgical outcomes were available for this patient, with grids over the right lateral frontal, right orbitofrontal, and right interhemispheric fissure, as well as with a depth electrode in the right lateral frontal region. Interictal activity was seen in the right lateral frontal grid and right frontal depth electrode but was also seen broadly in the other two grids. Seizures arose mainly from the right lateral frontal grid and depth electrode but also in the other regions almost simultaneously. The iEEG and MEG are therefore concordant, especially when considering the broad onset in the intracranial study and the MEG report. Although motor strip was involved, the patient had a right frontal lobectomy sparing motor strip which resulted in diagnosis of focal cortical dysplasia (FCD) type 2B. The patient was then seizure free for 2 years with recurrence in the motor region but has been seizure free on adjusted medication for 3 years since then. Figure 3 shows the MEG localizations (for both the EKM and HMM methods) and the postoperative MRI. The resected region clearly matches the area highlighted by MEG analysis.

Three further cases of patients with focal epilepsy with spike and wave are shown in Figures S3–S5, all with excellent agreement between the HMM and EKM. In these cases, spatial correspondence was 4 ± 1, 9 ± 5, and 3.9 ± 0.3 mm, and the temporal correspondence was 97% ± 4%, 100% ± 1%, and 96% ± 5%. In general, these results support our hypothesis that the HMM performs similarly to EKM in enabling the identification of epileptiform activity in time and space, at least in patients with focal spike and wave epileptiform activity. Spatiotemporal correspondence was high in all cases and data showed good correspondence across many runs, for each subject.

### 3.2 Case 2: Focal epilepsy with polymorphic activity

**FIGURE 4:**
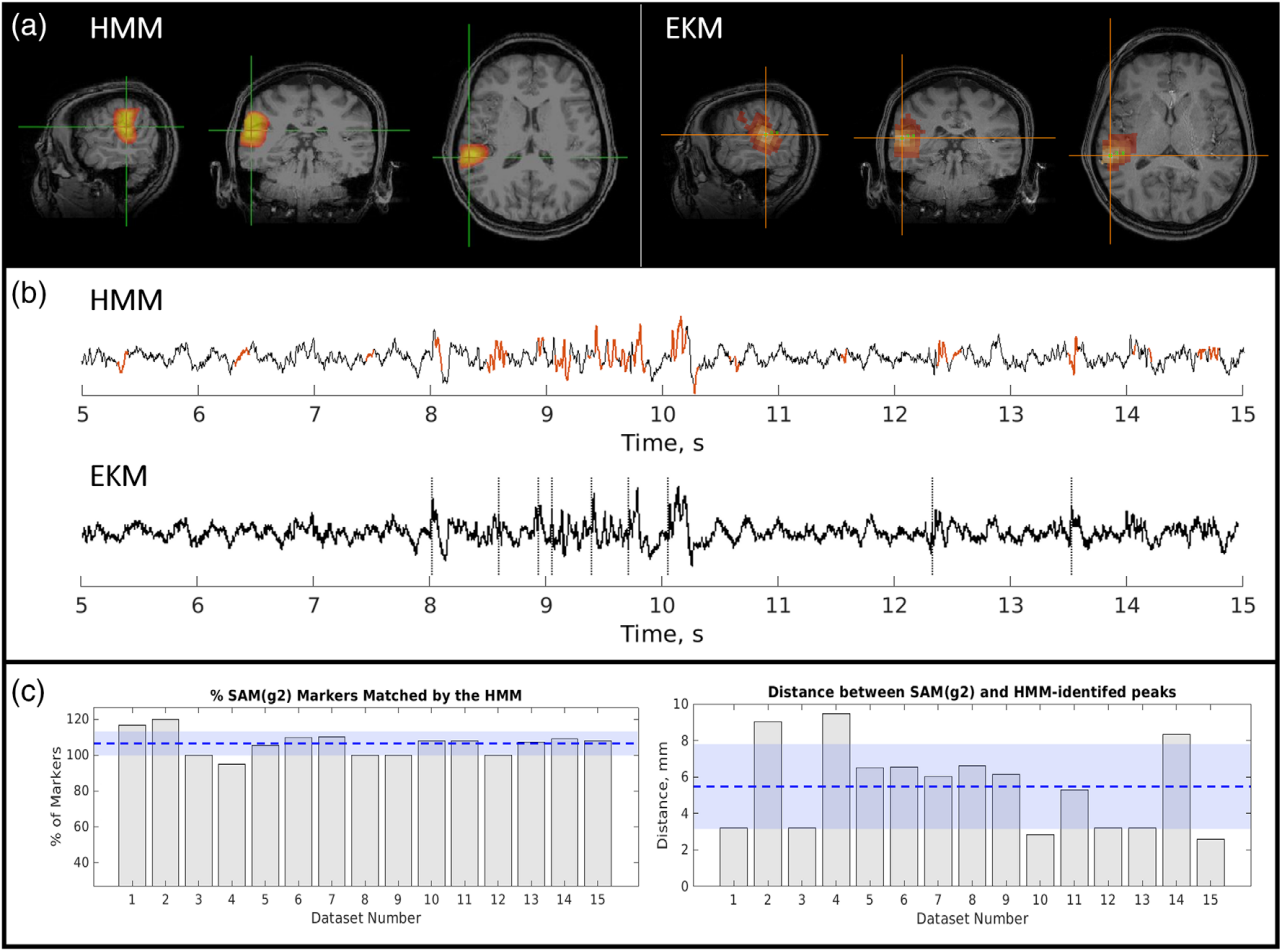
Epilepsy Case 2—Patient 5; focal, polymorphic bursts. (a) Left: hidden Markov model (HMM)‐derived map. Right: excess kurtosis mapping (EKM)‐derived map. Activity maps were thresholded to aid visual comparison. (b) Upper: Beamformer time course from the HMM peak; lower: Equivalent time course from the EKM peak. Dashed lines show time points of epileptogenic activity identified by EKM. Red regions show occurrences of the epileptiform state. (c) Left: Temporal coincidence of EKM markers with the epileptiform state. Right: Spatial correspondence

Case 2 (Figure 4) shows MEG data acquired in a patient with focal epilepsy, but without typical spikes in the MEG trace (Figure 4b). The patient's resting MEG data exhibited abundant polymorphic bursts of sharply contoured epileptiform activity, which (unlike spikes) change their temporal morphology on each occurrence. Such data are not amenable to conventional ECD source analysis; however, here we see that both the HMM and EKM generate a focal localization of the epileptogenic zone with excellent spatial agreement between the two methods. On average across fifteen 2‐min recordings, the spatial discrepancy between the HMM and EKM peak location was 5 ± 2 mm. In addition, the temporal coincidence of the epileptiform HMM state and the EKM‐derived markers was 106% ± 7%. The percentage of time when the HMM epileptiform state was active was 6% ± 3% (average and SD over all runs).

**FIGURE 5:**
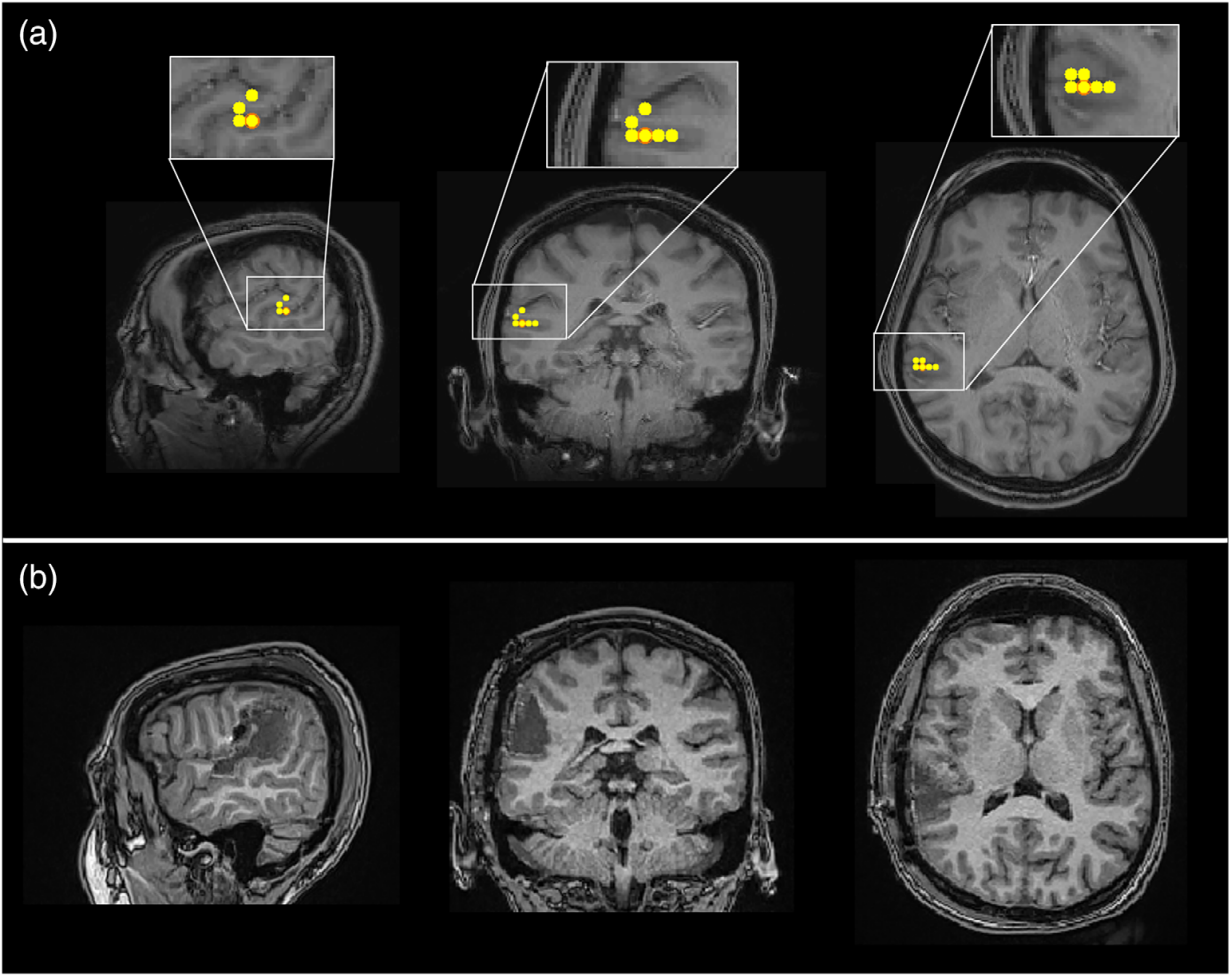
Epilepsy Case 2—magnetoencephalography (MEG) localizations compared with resected area for Patient 5 (focal polymorphic). Panel (a) shows the MEG localizations (peak virtual electrode locations) for the excess kurtosis mapping method (red markers) and the hidden Markov model method (yellow markers). Note that markers may be overlaid so that red markers are hidden under yellow ones. Panel (b) shows the resected area in the postoperative magnetic resonance imaging

iEEG for this patient was stereo‐EEG. Interictal activity arose mainly from the mid superior temporal gyrus (STG) electrode, as well as seizures, but there were rare interictal discharges in the angular gyrus as well. The patient had a focal resection of the posterior STG which resulted in pathology of FCD 2B. She has been seizure free since surgery (9 months) and has been able to reduce medication. The surgery did result in a conductive aphasia, but this has since improved. MEG was concordant in the post‐STG but also had some involvement in middle temporal gyrus (MTG) and right posterior insula which were not involved on stereo‐EEG. MEG localizations are shown in yellow and red markers (HMM and EKM, respectively) on the preoperative MRI alongside the resection zone on the postoperative MRI in Figure 5. There is close agreement between the identified MEG locations and the resected area.

A second case of focal epilepsy with polymorphic bursts is given in Figure S6; results again were similar with a spatial correspondence of 5 ± 3 mm and temporal correspondence of 113% ± 14%.

### 3.3 Case 3: Multifocal epilepsy with polymorphic activity

The above results show relatively straightforward cases of focal epilepsy, where abnormal epileptiform activity arises from a single location in the brain. However, in more complex cases, abnormal activity can occur from more than one location (often simultaneously), and in such cases, the challenge becomes determining where these multiple regions are, how they are related, and if possible, which region serves as the driver of an epileptiform network causing other areas to exhibit epileptiform activity.

**FIGURE 6:**
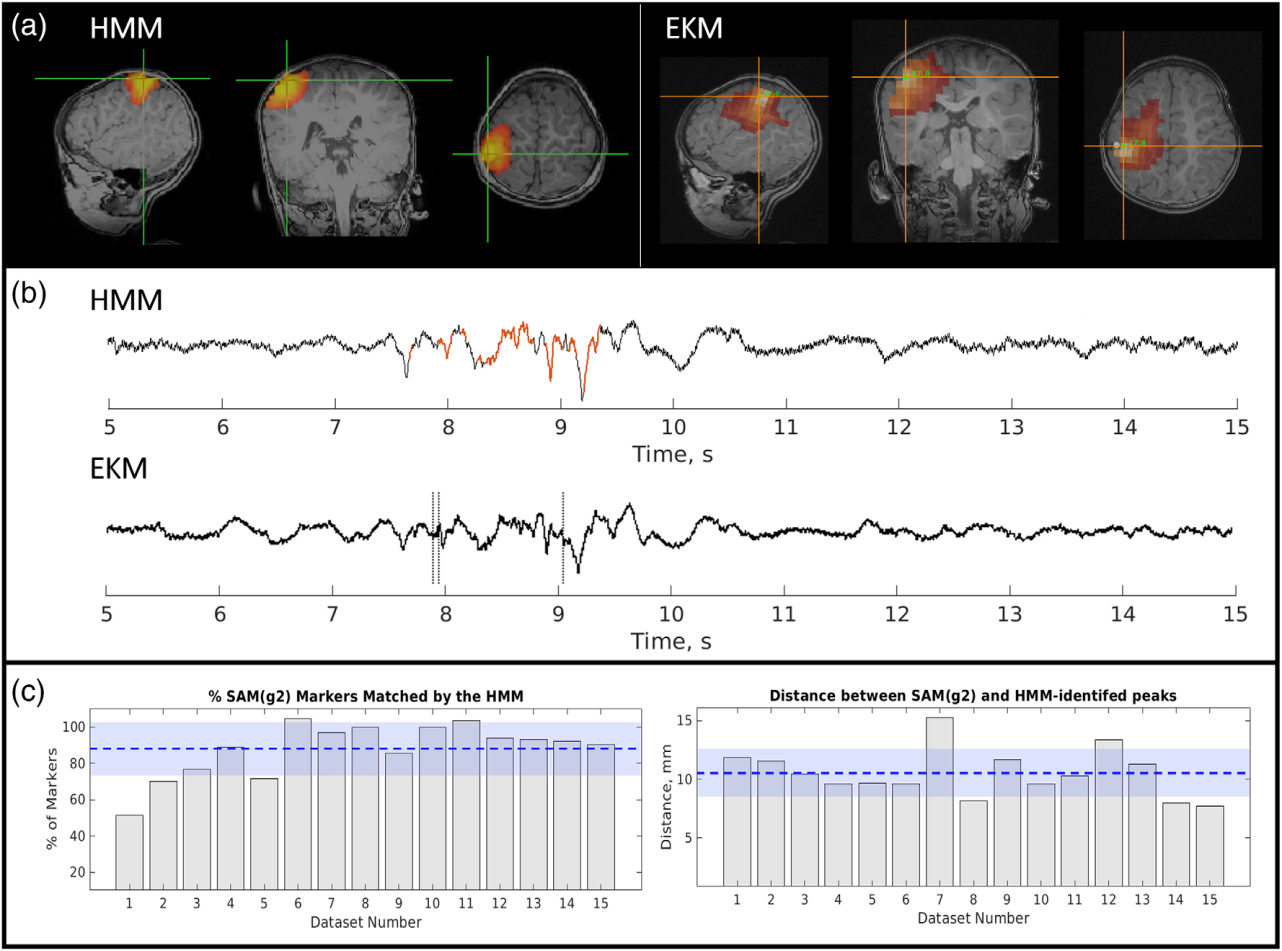
Epilepsy Case 3—Patient 8; multifocal, polymorphic. There was good agreement between methods for the spatial localization (see the maps for hidden Markov model [HMM; a, left] and excess kurtosis mapping [EKM; a, right]) with an average distance between HMM and EKM peaks of 11 ± 2 mm. The virtual depth electrode time courses for each method are shown in (b) with HMM state visits highlighted in red and EKM markers as dashed lines. The temporal match was also good with 88% ± 15% of EKM markers matched by an HMM state visit. The HMM state was active for 7% ± 3% of the total time

Case 3 is a patient with multifocal epilepsy; in total 15 datasets were acquired in this individual, results from a single representative run are shown in Figure 6. This patient exhibited EKM and HMM peaks in right periorolandic areas. For 11/15 runs, there was just one epileptiform state describing the activity from these regions, in the other four runs two epileptiform states were identified. Where more than one HMM state was identified, there was no obvious temporal relationship between them.

**FIGURE 7:**
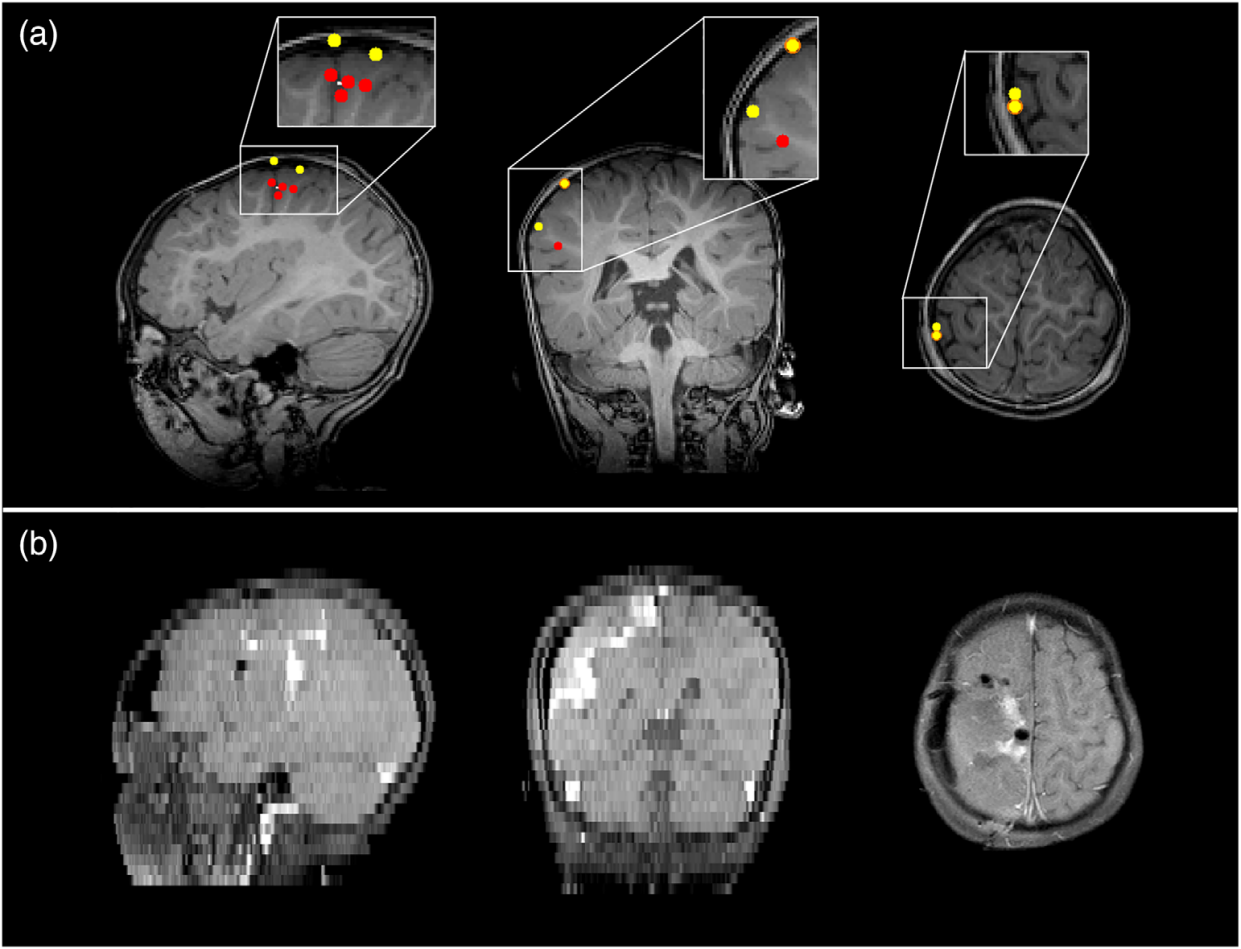
Epilepsy Case 3—magnetoencephalography (MEG) localizations compared with resected area for Patient 8 (multifocal polymorphic). Panel (a) shows The MEG localizations for the EKM method (red markers) and the hidden Markov model method (yellow markers). Panel (b) shows the resected area in the postoperative magnetic resonance imaging (MRI). Note that the postoperative MRI has different resolutions in the sagittal, coronal, and axial planes

In this patient, iEEG was grids and strips over the right superior and inferior frontoparietal region and right subtemporal area. Interictal activity and seizures arose in the right superior frontoparietal grid with rare sharps in the inferior grid. Seizures had onset in the superior frontoparietal grid but also more broadly at times in the superior frontoparietal grid and a small area of the inferior parietal grid. The patient underwent a right frontal and anterior parietal resection which resulted in a left hemiparesis and visual field cut, but has been seizure free since surgery (5.5 years) and is now off medications. Pathology was also FCD 2B. MEG was concordant in the right perirolandic region but also showed something in the right occipital and right hippocampal tail that were not covered by the implant well enough to establish concordance. Preoperative and postoperative MRIs are shown in Figure 7 for this patient. MEG localizations are shown for the HMM and EKM methods (yellow and red markers, respectively). The postoperative MRI (Figure 7b) shows regions of the brain which were resected.

### 3.4 Case 4: Multifocal epilepsy with polymorphic activity

**FIGURE 8:**
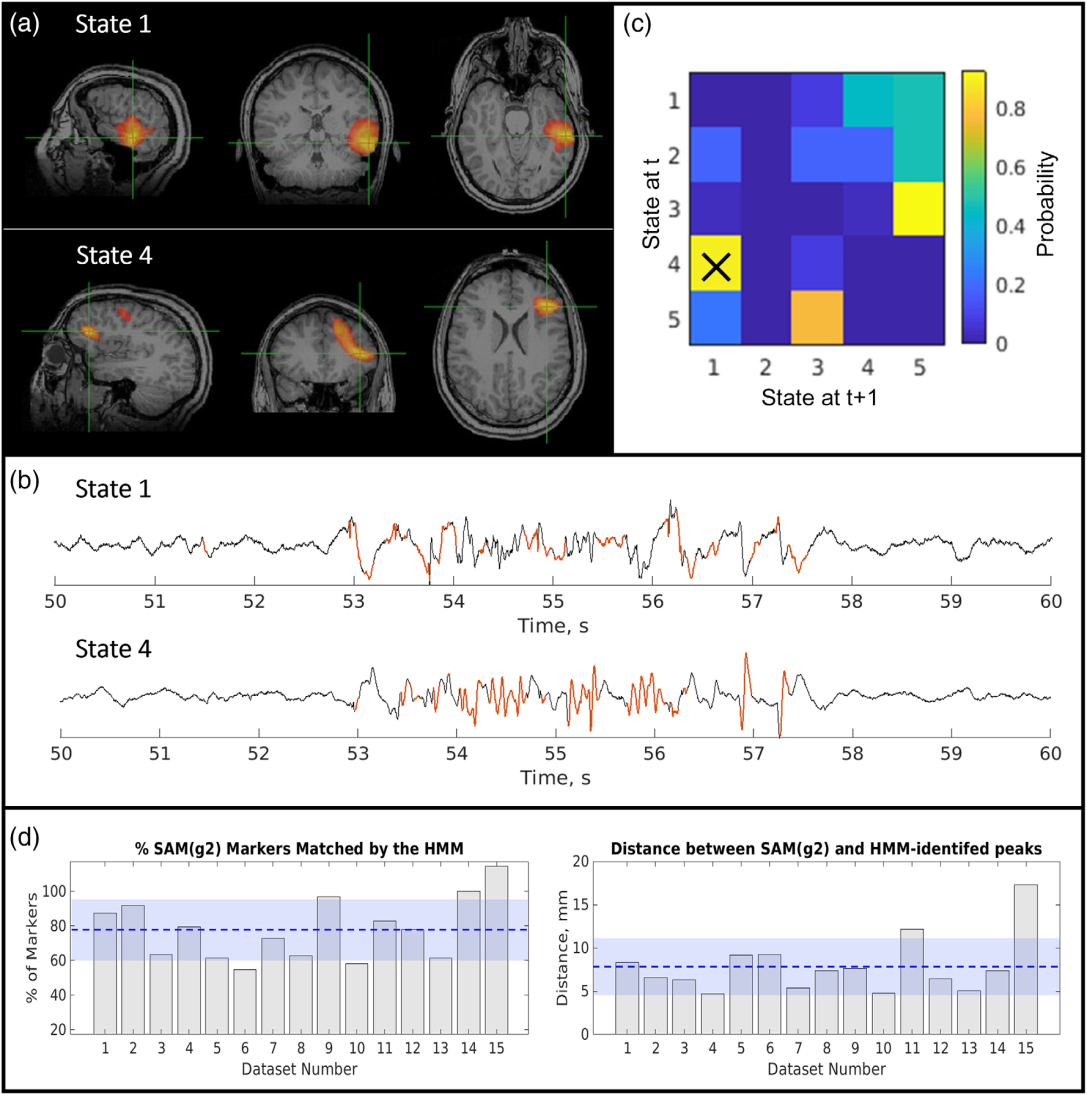
Epilepsy Case 4—Patient 10; multifocal, polymorphic bursts. (a) Upper: hidden Markov model (HMM)‐derived map from State 1. Lower: HMM‐derived map from State 4. (b) Upper: Beamformer time course from the HMM peak for State 1; lower: Equivalent time course from the HMM peak for State 4. Note both states show epileptiform activity. (c) State transition matrix. Elements represent the probability of a transition between states; for example, element (2, 1) would represent the probability of a transition from State 2 to State 1; element (1, 2) would represent the probability of a transition from State 1 to State 2. Panels (a–c) show the result from a single representative run. The average spatial and temporal correspondence between methods is shown in (d) with 78% ± 18% of excess kurtosis mapping markers matched by an HMM state visit and an average Euclidian distance of 8 ± 3 mm between peaks. The amount of time spent in the epileptiform state was 3% ± 1% of the total time

Case 4 is another patient with multifocal polymorphic activity arising from both the left frontal and left temporal lobes. The HMM consistently separated the activity from these regions out into distinct states (shown in Figure 8). Interestingly, examination of state transition probabilities demonstrated that State 1 (left temporal) had an 85% likelihood of being preceded in time by State 4 (left frontal); in other words, a polymorphic burst in the frontal lobe was almost always followed by a polymorphic burst in the temporal lobe. Potentially, this suggests that the frontal lobe region is the driving signal and root cause of the epileptiform activity observed in the temporal lobe.

Across all 15 runs in this same subject, 10/15 allowed identification of 2 epileptiform states (including the run shown in Figure 4); 3/15 runs had 3 epileptiform states, and 2 runs had just 1 epileptiform state. Of the 13 datasets with more than one epileptiform state, 9/13 had a clear transition from the frontal to the temporal locations (transition probability 84% ± 9%; mean and SD across these 9 runs). The other four had no clear transitions between states. The percentage of time which the states were active was 3% ± 1% and 4% ± 1% for the temporal and frontal epileptiform states, respectively.

We also compared the results shown in Figure 8 with the output of our EKM algorithm. EKM generated a single map which also had peaks in frontal and temporal lobe. On average (across 15 runs) the mean spatial correspondence for the frontal lobe peak was 8 ± 3 mm and the equivalent distance for the temporal lobe peak was 7 ± 4 mm, once again implying spatial correspondence between the HMM and EKM. This is impressive given the challenges posed by such a complicated case to each of these methods. It is worth noting that although the EKM method places temporal markers in the data to help epileptologists assess whether a spike in one part of the brain precedes a spike in another area, the HMM provides additional information about the temporal relationship between the two brain locations using the state transition probabilities. Furthermore, the HMM uses all of the state data to estimate transitions, something which becomes particularly important in cases without clear spikes. No invasive assessment data or surgical outcomes are available for this patient.

Two further cases of multifocal epilepsy are presented in Figures S7 and S8. In both cases, results are similar to those shown in Figures 6 and 8.

### 3.5 Group results

For each of the three patient groups, the average spatial and temporal correspondence between the two methods was found over subjects and this is shown in Table 2. There was good agreement between the two methods for all three groups. This is especially encouraging given the complexity of the multifocal patients, with less than a centimeter discrepancy between the peak locations. There was more variation in the temporal coincidence metric which is likely to be because there were fewer EKM markers in the multifocal polymorphic data because the amplitude of the virtual electrode data rarely exceeded the threshold needed for marker placement. This meant that if the HMM missed a single marker, it resulted in a much‐reduced percentage coincidence.

**TABLE 2:** Group results

	Average temporal coincidence (±SD over subjects; %)	Average spatial correspondence (±SD across subjects; mm)
Focal spike and wave	99 ± 3	8 ± 5
Focal polymorphic	110 ± 5	5.45 ± 0.03
Multifocal polymorphic	80 ± 5	8 ± 2

## 4 DISCUSSION

Epilepsy is a debilitating disorder in which both symptoms and treatments differ markedly across patients. In some cases where pharmacological intervention fails to control seizures, patients become candidates for surgery in which affected brain regions are resected. For many patients, such intervention offers seizure freedom (and thus a marked improvement in quality of life). However, success greatly depends on presurgical planning to accurately identify the epileptogenic region(s) and the current clinical pathway (involving multidisciplinary team input with interictal and ictal EEG ± iEEG, and MRI) is not always successful in identifying candidate regions for resection (or indeed implantation of the iEEG). Consequently, improvements to this pathway could enable more patients to become eligible for surgery and might offer improved outcomes for those who do have surgery.

MEG has a significant and established role in epilepsy, offering high‐precision mapping of epileptogenic and eloquent cortex (Kim et al., 2013; Rampp et al., 2019; Schwartz et al., 2010). However, the current methods for analysis of MEG data are limited. ECD—still the most widely used technique—is unsuited to multifocal epilepsies, and cases where temporal morphology of interictal events fails to include isolated high amplitude spikes. Some of the limitations of ECD are lifted via the use of kurtosis‐based techniques; however, these too offer limited sensitivity; they only respond to activity that is characterized by non‐Gaussianity, and in multifocal cases, EKM provides limited information about the relationship between brain regions. This means that identified brain areas can be erroneously discarded due to a lack of evidence that they are involved in an epileptic network. Consequently, there is room for more adaptable processing pipelines to analyze MEG data in epilepsy patients.

In this work, we have demonstrated that an HMM is a promising technique to localize epileptiform activity in both time and space. The HMM has been used increasingly in the analysis of MEG data in recent years, to elucidate the complex spatiotemporal dynamics of brain networks (Baker et al., 2014; Seedat et al., 2020; Vidaurre et al., 2018; Woolrich et al., 2013). Here we show that, by seeking short segments of data with unique spatiospectral characteristics, the HMM allows automatic detection of epileptiform activity. Importantly, the HMM does not rely on a single data characteristic—for example, high amplitude spikes, or a non‐Gaussian statistical distribution. In addition, activity does not have to repeat in time (like a template). Rather, the HMM employs features of the data (e.g., sharp contouring) that can vary on every occurrence of an epileptiform state. Consequently, as we have demonstrated, it can adapt to the heterogeneity of the disorder; the same algorithm will find spike and waves, sharps, or polymorphic bursts with equal efficiency. The flexible, data‐driven approach of the HMM lends itself naturally to personalized medicine, where the output of the model will be specific to each patient.

We compared the output of our HMM to the more established EKM technique (which is embedded in a number of clinical pathways for epilepsy across multiple centers). Spatial agreement was within 8 ± 3 mm (mean and SD across all subjects and runs in all three patient groups), showing that epileptic foci could be identified using the HMM with high spatial acuity. Temporal agreement was also good, with 94% ± 13% (mean over all subjects in all patient groups) of epileptiform events detected by the standard EKM pipeline matched by the occurrence of the epileptiform state, identified by the HMM. Importantly, whilst the HMM looks for a specific spatiospectral pattern, the EKM pipeline is only able to find the timing of epileptic bursts via the use of a threshold: specifically, the EKM threshold is set by some arbitrary parameter (6) multiplied by the RMS amplitude. Thus, epileptiform activity with lower amplitude is likely to be missed by this overly simplified technique. The HMM on the other hand relies on the occurrence of a specific multivariate spatiospectral pattern. Consequently, it is less likely to miss epileptiform bursts.

The HMM has demonstrated advantages when looking at multifocal epilepsy cases. In such complex cases, ECD is limited since one must make an a priori decision on the number of active foci, which is always unknown. EKM is better, since it allows identification of multiple regions, and the manual comparison of kurtosis markers can identify whether one sharply contoured event from one location consistently precedes another. Similarly, the HMM can identify multiple epileptiform foci but provides more information regarding the temporal relationship between the identified regions. Specifically, where multiple foci appear within a single state, then those regions (by definition) are related temporally, that is, the regions are “active” together. This was seen in Patient 9 (Figure S8) in which (in 6/13 cases) an epileptogenic focus close to an area of resected tissue in the frontal lobe appeared alongside a second peak in parietal cortex. The signals from the second peak were not epileptiform and thus had initially been dismissed; however, the fact that the activity generated increases in variance in synchrony with the peak close to the resection, indicates that it is likely a part of the epileptic network. Thus, simply by virtue of the epileptiform state containing two peaks, we gain more information than we would with an equivalent image acquired using EKM. This could be particularly advantageous in patients with, for example, tuberous sclerosis were identifying a network of epileptiform activity involving multiple tubers could inform surgical planning.

Similarly in Case 4 (Figure 8), we discovered two states containing epileptiform activity. In this case, each state contained only one region. However, the states demonstrated a temporal dependence according to the transition probability matrix; specifically, a burst of activity in temporal lobe was almost always preceded by a similar burst in frontal lobe. This again is an example of how the additional information afforded by the HMM might be used to provide valuable information about an epileptic network. A limitation of our study is that iEEG data were not available for either of these subjects in these specific locations, making it challenging to verify the MEG results. Future studies should seek to replicate this result in patients with iEEG so that the MEG localizations and transitions can be verified against the current clinical gold standard. However, it should be noted that iEEG is heavily reliant on the presurgical hypothesis and so is only reliable if the hypothesis was correct and the electrodes covered the epileptogenic zone. Nevertheless, the evidence presented not only demonstrates localization of epileptiform activity, but also serves as an interesting example of the how the HMM may offer additional information—beyond that gained from the existing EKM approach and warrants further investigation in a larger cohort of patients which covers a breadth of epilepsy types and includes those who have undergone either implantation of electrodes for iEEG or surgery so that postsurgical outcomes can be used as a ground truth to assess against.

The HMM method implemented in this study is designed to be a semiautomated spike‐detection algorithm to aid clinicians in quickly and efficiently identifying epileptiform discharges in temporal data as well as localizing the regions of the brain in which they arise. We anticipate that in the clinic, there will be two manual processing steps required: the first is the visual inspection of data to remove those datasets with excessive head motion. This could be easily undertaken by somebody without training in reading clinical MEG data or identifying epileptiform discharges and could potentially be automated (at least in part). The second step will be to interpret the output of the HMM to select the epileptiform state(s)—this will likely require the assistance of a trained epileptologist. This method utilizes both the power of machine learning (with the HMM) and clinical expertise, but there is the potential to further automate the pipeline by employing additional machine learning techniques to learn features of epileptiform states for automatic state selection in the future.

There are a number of further extensions to the present methodology which should be considered. First, the current version of the model operates using sensor space data, and localization is achieved using a beamformer. It is theoretically possible to apply the beamformer first, and then the HMM, and this may offer advantages: For example, the beamformer is known to supress fields from non‐brain sources, which would mean a source space HMM is less likely to be biased by interference. However, it is also significantly more computationally demanding since the HMM must be run on many thousands of (voxel‐based) signals rather than a few hundred channels. This was found to be impractical with current computing power, and whilst brain parcellation (e.g., dividing the brain into a smaller number of anatomically [or functionally] meaningful regions) offers a means to reduce dimensionality, it also reduces spatial specificity which is of key importance in this application. Thus, we feel the method presented is the most practical currently, but the use of better computing may ultimately enable a source space HMM. In addition, although we have applied our method to MEG data, application to EEG data or concurrent EEG/MEG data would also be valuable. In the former case, the limitations surrounding the spatial resolution of EEG would mean low spatial specificity, but nevertheless, the HMM might offer a useful means to automatically identify time points which contain interictal events—this could enable a significant saving in time for epileptologists who review EEG data. For concurrent EEG/MEG, there is some evidence that the combination increases sensitivity (e.g., to radial sources) This may offer a significant advantage over MEG alone. It should also be noted that the data used in this study were from a single site and acquired using one type of MEG system (CTF)—future studies should test the reliability of the HMM optimized with these data at other sites.

The advent of wearable MEG systems, based on optically pumped magnetometer detection of the neuromagnetic field (Boto et al., 2019; Hill et al., 2019) may also offer significant advantages. OPMs measure magnetic fields much closer to the brain than SQUIDs (superconducting quantum interference devices), offering greater spatial precision and sensitivity. Likewise, bespoke helmets, which can be removed and replaced, with sensors going back to the same locations relative to brain anatomy each time, may offer the opportunity to record longer datasets. This would undoubtedly create a more accurate model which could help to overcome the variations in HMM output over multiple runs. Wearable MEG would also enable free movement whilst scanning; one of the key limitations of our study is that seven of the patients were under general anesthesia to stop them moving. Unfortunately, anesthesia (even lightly applied) is likely to reduce all neural activity including epileptiform activity, and so scanning patients whilst conscious is desirable. Working with child life specialists to prepare patients for MEG scanning, using a mock scanner, and other methods, including a wearable MEG system, could all be ways to either get patients to stay still or create a scanning environment where they no longer have to keep completely still, rendering anesthesia unnecessary in most cases. Finally, whilst in this article, we concentrate mainly on spikes, sharps and polymorphic bursts, there are other forms of atypical signaling (e.g., fast ripples [>200 Hz] and slowing [1–4 Hz]) which are consistently observed in patients with epilepsy. Unlike ECD or EKM, the HMM could readily be extended to focus on these spectral features. It would be interesting to see how the HMM performs in a larger patient cohort and further studies should include patients with these atypical waveforms as well as a wider range of epilepsy types (outside of fronto/temporal epilepsy).

## 5 CONCLUSION

The HMM offers an alternative to ECD and EKM for identifying and localizing epileptiform activity in the human brain. In 10 subjects, we found good spatial agreement between methods, with the HMM able to provide localization which matched, within a few mm, that from EKM. Moreover, we found that the HMM offers more information about epileptiform activity, particularly in multifocal cases. As the use of MEG continues to grow in epilepsy, particularly given the advent of new, cheaper, and more practical MEG systems, clinical pathways should look to exploit the HMM, and related techniques, as a means of spatiotemporal mapping of epileptogenic cortex. This would aid identification of patients suitable for epilepsy surgery with the goal of subsequent seizure freedom (or significant seizure reduction) resulting in huge benefit to quality of life.

## Supporting information


**Appendix S1** Supporting Information

## Data Availability

Data available on request due to privacy/ethical restrictions.

## References

[hbm26118-bib-0001] Agirre‐Arrizubieta, Z. , Thai, N. J. , Valentin, A. , Furlong, P. L. , Seri, S. , Selway, R. P. , Elwes, R. B. C. , & Alarcon, G. (2014). The value of magnetoencephalography to guide electrode implantation in epilepsy. Brain Topography, 27, 197–207. 10.1007/s10548-013-0330-x 24249204

[hbm26118-bib-0002] Bagić, A. I. , Knowlton, R. C. , Rose, D. F. , & Ebersole, J. S. (2011). American clinical magnetoencephalography society clinical practice guideline 1: Recording and analysis of spontaneous cerebral activity. Journal of Clinical Neurophysiology, 28(4), 348–354. 10.1097/WNP.0b013e3182272fed 21811121

[hbm26118-bib-0003] Baker, A. P. , Brookes, M. J. , Rezek, I. A. , Smith, S. M. , Behrens, T. , Smith, P. J. P. , & Woolrich, M. (2014). Fast transient networks in spontaneous human brain activity. eLife, 3, e01867. 10.7554/eLife.01867 24668169 PMC3965210

[hbm26118-bib-0004] Beghi, E. , Giussani, G. , & Collaborators, G. E. (2019). Global, regional, and national burden of epilepsy, 1990–2016: A systematic analysis for the global burden of disease study 2016. The Lancet Neurology, 18(4), 357–375. 10.1016/S1474-4422(18)30454-X 30773428 PMC6416168

[hbm26118-bib-0005] Boto, E. , Hill, R. M. , Rea, M. , Holmes, N. , Seedat, Z. A. , Leggett, J. , Shah, V. , Osborne, J. , Bowtell, R. , & Brookes, M. J. (2021). Measuring functional connectivity with wearable MEG. NeuroImage, 230, 117815. 10.1016/j.neuroimage.2021.117815 33524584 PMC8216250

[hbm26118-bib-0006] Boto, E. , Holmes, N. , Leggett, J. , Roberts, G. , Shah, V. , Meyer, S. S. , Muñoz, L. D. , Mullinger, K. J. , Tierney, T. M. , Bestmann, S. , Barnes, G. R. , Bowtell, R. , & Brookes, M. J. (2018). Moving magnetoencephalography towards real‐world applications with a wearable system. Nature, 555, 657–661. 10.1038/nature26147 29562238 PMC6063354

[hbm26118-bib-0007] Boto, E. , Seedat, Z. A. , Holmes, N. , Leggett, J. , Hill, R. M. , Roberts, G. , Shah, V. , Fromhold, T. M. , Mullinger, K. J. , Tierney, T. M. , Barnes, G. R. , Bowtell, R. , & Brookes, M. J. (2019). Wearable neuroimaging: Combining and contrasting magnetoencephalography and electroencephalography. NeuroImage, 201, 116099. 10.1016/j.neuroimage.2019.116099 31419612 PMC8235152

[hbm26118-bib-0008] Brookes, M. J. , Leggett, J. , Rea, M. , Hill, R. M. , Holmes, N. , Boto, E. , & Bowtell, R. (2022). Magnetoencephalography with optically pumped magnetometers (OPM‐MEG): The next generation of functional neuroimaging. Trends in Neurosciences, 1824, 621–634. 10.1016/j.tins.2022.05.008 PMC10465236 35779970

[hbm26118-bib-0031] Brookes, M. J. , et al. (2008). Simultaneous EEG source localisaion and artifact rejection during concurrent fMRI by means of spatial filtering. NeuroImage 40(3), 1090–1104. 10.1016/j.neuroimage.2007.12.030 18296069

[hbm26118-bib-0009] Ebersole, J. S. (1997). Magnetoencephaography/magnetic source imaging in the assessment of patients with epilepsy. Epilepsia, 38(4), S1–S5. 10.1111/j.1528-1157.1997.tb04533.x 9240234

[hbm26118-bib-0010] Gaetz, W. , Gordon, R. S. , Papadelis, C. , Fujiwara, H. , Rose, D. F. , Edgar, J. C. , Schwartz, E. S. , & Roberts, T. P. L. (2015). Magnetoencephalography for clinical pediatrics: Recent advances in hardware, methods, and clinical applications. Journal of Pediatric Epilepsy, 4(4), 139–155. 10.1055/s-0035-1563726

[hbm26118-bib-0011] Gofshteyn, J. S. , Le, T. , Kessler, S. , Kamens, R. , Carr, C. , Gaetz, W. , Bloy, L. , TPL, R. , Schwartz, E. S. , & Marsh, E. D. (2019). Synthetic aperture magnetometry and excess kurtosis mapping of magnetoencephalography (MEG) is predictive of epilepsy surgical outcome in a large pediatric cohort. Epilepsy research, 155, 106151. 10.1016/j.eplepsyres.2019.106151 31247475 PMC6699633

[hbm26118-bib-0012] Hall, M. , Nissen, I. A. , van Straaten, E. , Furlong, P. L. , Witton, C. , Foley, E. , Seri, S. , & Hillebrand, A. (2018). An evaluation of kurtosis beamforming in magnetoencephalography to localize the epileptogenic zone in drug resistant epilepsy patients. Clinical Neurophysiology, 129(6), 1221–1229. 10.1016/j.clinph.2017.12.040 29660580 PMC5953276

[hbm26118-bib-0013] Higgins, C. , Liu, Y. , Vidaurre, D. , Kurth‐Nelson, Z. , Dolan, R. , Behrens, T. , & Woolrich, M. (2021). Replay bursts in humans coincide with activation of the default mode and parietal alpha networks. Neuron, 109(5), 882–893. 10.1016/j.neuron.2020.12.007 33357412 PMC7927915

[hbm26118-bib-0014] Hill, R. M. , Boto, E. , Holmes, N. , Hartley, C. , Seedat, Z. A. , Leggett, J. , Roberts, G. , Shah, V. , Tierney, T. M. , Woolrich, M. W. , Stagg, C. J. , Barnes, G. R. , Bowtell, R. , Slater, R. , & Brookes, M. J. (2019). A tool for functional brain imaging with lifespan compliance. Nature Communications, 10, 4785. 10.1038/s41467-019-12486-x PMC6831615 31690797

[hbm26118-bib-0015] Hill, R. M. , Boto, E. , Rea, M. , Holmes, N. , Leggett, J. , Coles, L. A. , Papastavrou, M. , Everton, S. K. , BAE, H. , Sims, D. , Osborne, J. , Shah, V. , Bowtell, R. , & Brookes, M. J. (2020). Multi‐channel whole‐head OPM‐MEG: Helmet design and a comparison with a conventional system. NeuroImage, 219, 116995. 10.1016/j.neuroimage.2020.116995 32480036 PMC8274815

[hbm26118-bib-0016] Kim, H. , Chung, C. K. , & Hwang, H. (2013). Magnetoencephalography in pediatric epilepsy. Clinical and Experimental Pediatrics, 56(10), 431–438. 10.3345/kjp.2013.56.10.431 PMC3827491 24244211

[hbm26118-bib-0017] Mohan, M. , Keller, S. , Nicolson, A. , Biswas, S. , Smith, D. , Farah, J. O. , Eldridge, P. , & Wieshmann, U. (2018). The longterm outcomes of epilepsy surgery. PLoS One, 13(5), e0196274. 10.1371/journal.pone.0196274 29768433 PMC5955551

[hbm26118-bib-0018] Murakami, H. , Wang, Z. I. , Marashly, A. , Krishnan, B. , Prayson, R. A. , Kakisaka, Y. , Mosher, J. C. , Bulacio, J. , Gonzalez‐Martinez, J. A. , Bingaman, W. E. , Najm, I. M. , Burgess, R. C. , & Alexopoulos, A. V. (2016). Correlating magnetoencephalography to stereo‐electroencephalography in patients undergoing epilepsy surgery. Brain, 139(11), 2935–2947. 10.1093/brain/aww215 27567464 PMC5091043

[hbm26118-bib-0019] Nissen, I. A. , Stam, C. J. , Citroen, J. , Reijneveld, J. C. , & Hillebrand, A. (2016). Preoperative evaluation using magnetoencephalography: Experience in 382 epilepsy patients. Epilepsy Research, 124, 23–33. 10.1016/j.eplepsyres.2016.05.002 27232766

[hbm26118-bib-0020] Quinn, A. J. , Vidaurre, D. , Abeysuriya, R. , Becker, R. , Nobre, A. C. , & Woolrich, M. W. (2018). Task‐evoked dynamic network analysis through hidden Markov modeling. Frontiers in Neuroscience, 12, 603. 10.3389/fnins.2018.00603 30210284 PMC6121015

[hbm26118-bib-0021] Rampp, S. , Stefan, H. , Wu, X. , Kaltenhäuser, M. , Maess, B. , Schmitt, F. C. , Wolters, C. H. , Hamer, H. , Kasper, B. S. , Schwab, S. , Doerfler, A. , Blümcke, I. , Rössler, K. , & Buchfelder, M. (2019). Magnetoencephalography for epileptic focus localization in a series of 1000 cases. Brain, 142(10), 3059–3071. 10.1093/brain/awz231 31373622

[hbm26118-bib-0022] Robinson, S. E. , Nagarajan, S. S. , Mantle, M. , Gibbons, V. , & Kirsch, H. (2004). Localization of interictal spikes using SAM(g2) and dipole fit. Neurology & Clinical Neurophysiology, 74. PMC4041981 16012648

[hbm26118-bib-0030] Robinson, S. E. and Vrba J. (1999). Functional neuroimaging by synthetic aperture magnetometry. Recent Advances in Biomagnetism: 302–305.

[hbm26118-bib-0023] Schwartz, E. S. , Edgar, J. C. , Gaetz, W. C. , & Roberts, T. P. L. (2010). Magnetoencephalography. Pediatric Radiology, 40, 50–58. 10.1007/s00247-009-1451-y 19937237

[hbm26118-bib-0024] Seedat, Z. A. , Quinn, A. J. , Vidaurre, D. , Liuzzi, L. , Gascoyne, L. E. , Hunt, B. A. E. , O'Neill, G. C. , Pakenham, D. O. , Mullinger, K. J. , Morris, P. G. , Woolrich, M. W. , & Brookes, M. J. (2020). The role of transient spectral ‘bursts’ in functional connectivity: A magnetoencephalography study. NeuroImage, 209, 116537. 10.1016/j.neuroimage.2020.116537 31935517

[hbm26118-bib-0025] Stefan, H. , Wu, X. , Buchfelder, M. , Rampp, S. , Kasper, B. , Hopfengartner, R. , Schmitt, F. , Dörfler, A. , Blümcke, I. , Zhou, D. , & Weigel, D. (2011). MEG in frontal lobe epilepsies: Localization and postoperative outcome. Epilepsia, 52(12), 2233–2238. 10.1111/j.1528-1167.2011.03265.x 21933178

[hbm26118-bib-0026] Tang, L. , Mantle, M. , Ferrari, P. , Schiffbauer, H. , Rowley, H. A. , Barbaro, N. M. , Berger, M. S. , & Roberts, T. P. L. (2003). Consistency of interictal and ictal onset localization using magnetoencephalography in patients with partial epilepsy. Journal of Neurosurgery, 98(4), 837–845. 10.3171/jns.2003.98.4.0837 12691410

[hbm26118-bib-0027] Vidaurre, D. , Hunt, L. T. , Quinn, A. J. , Hunt, B. A. E. , Brookes, M. J. , Nobre, A. C. , & Woolrich, M. W. (2018). Spontaneous cortical activity transiently organises into frequency specific phase‐coupling networks. Nature Communications, 9, 2987. 10.1038/s41467-018-05316-z PMC6065434 30061566

[hbm26118-bib-0028] Wheless, J. W. , Willmore, L. J. , Breier, J. I. , Kataki, M. , Smith, J. R. , King, D. W. , Meador, K. J. , Park, Y. D. , Loring, D. W. , Clifton, G. L. , Baumgartner, J. , Thomas, A. B. , Constantinou, J. E. , & Papanicolaou, A. C. (1999). A comparison of magnetoencephalography, MRI, and V‐EEG in patients evaluated for epilepsy surgery. Epilepsia, 40(7), 931–941. 10.1111/j.1528-1157.1999.tb00800.x 10403217

[hbm26118-bib-0029] Woolrich, M. W. , Baker, A. P. , Luckhoo, H. , Mohseni, H. , Barnes, G. R. , Brookes, M. J. , & Rezek, I. A. (2013). Dynamic state allocation for MEG source reconstruction. NeuroImage, 77, 77–92. 10.1016/j.neuroimage.2013.03.036 23545283 PMC3898887

