# Supplementary material for: Mapping Interictal activity in epilepsy using a hidden Markov model: A magnetoencephalography study
Source: Hum Brain Mapp. 2022 Oct 19;44(1):66–81. doi: 10.1002/hbm.26118 (PMC9783449; doi:10.1002/hbm.26118)
Supplement: Supplementary file 1 — Appendix S1 Supporting Information [file HBM-44-66-s001.docx]

**Supplementary Information**

**Choice of HMM Model Parameters**

**
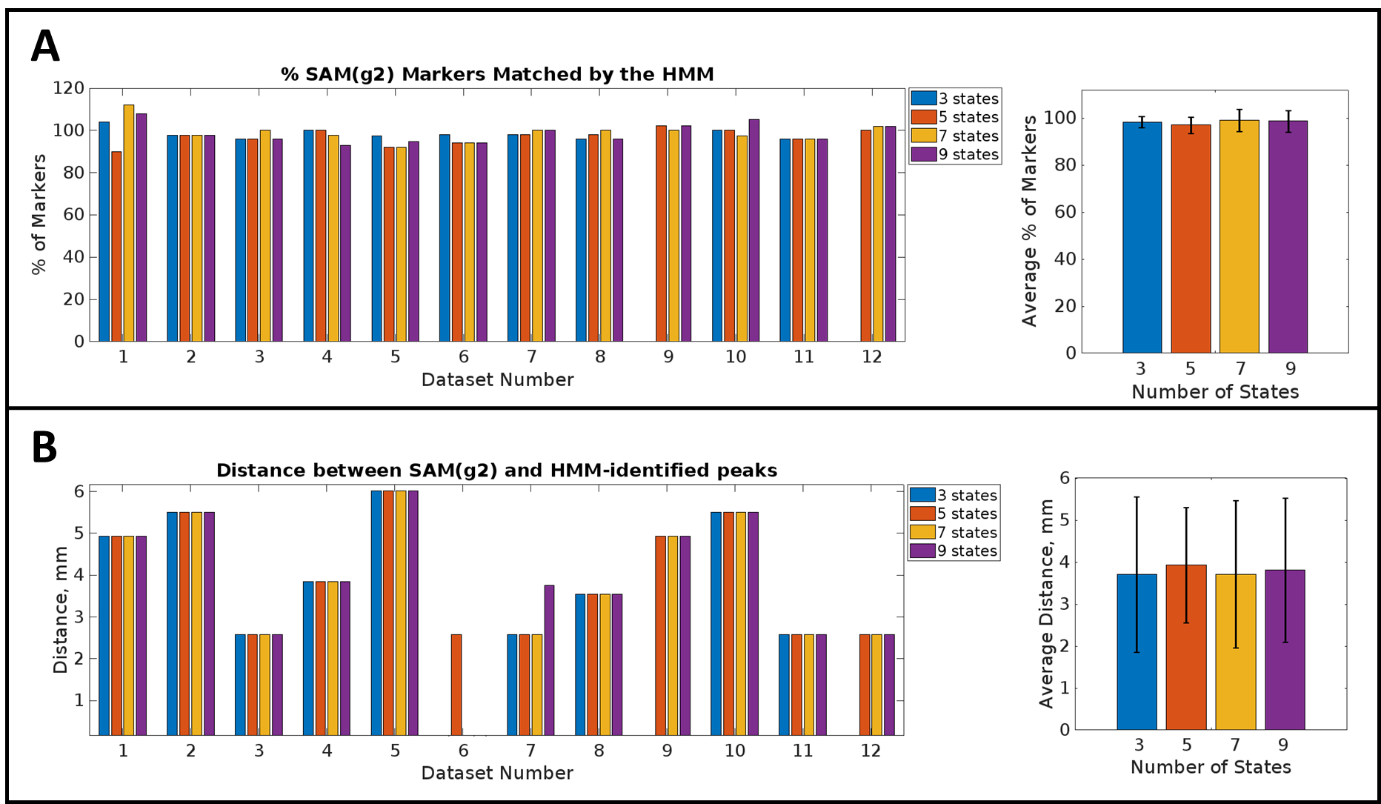
**To assess the impact of the choice of model parameters used for the HMM inference, different values for the number of states and lag durations were computed in one patient and the resulting temporal and spatial localisations of the interictal activity were compared with EKM. It is important to note that in this case EKM simply served as a reference point which we could compare our HMM localisations to, rather than acting as a gold standard method for localising epileptiform discharges. The number of states was varied - 3, 5, 7 and 9 states were used with no significant difference in the model output between them (see Figure S1). This is reassuring as it shows that the state characterisations were robust to changes in the number of states. However, varying the duration of the time-delay embeddings did change the localisation results (see Figure S2). This makes sense because states are characterised on their spectral content and a time window of longer duration will change the type of activity the HMM focuses on to slower frequencies, rather than the sharp components of the neuronal activity. The lag durations used in this paper (73ms) to calculate the state autocovariance matrices captures a high percentage of the EKM markers and results in a small difference in the spatial localisations of the two techniques (<5mm).

***Figure S1:*** *Varying the number of states used for model inference does not significantly change the result of the HMM temporal or spatial localisations for the epileptiform state in patient 1. Panel A shows the temporal match of the HMM epileptiform state visits with the EKM markers for each two-minute dataset (left) and for the average over all datasets (right). Panel B shows the spatial match of the HMM epileptiform state and SAM(g2) localisations for all datasets (left) and the average over datasets (right). The error bars show the standard deviation over datasets.*

***
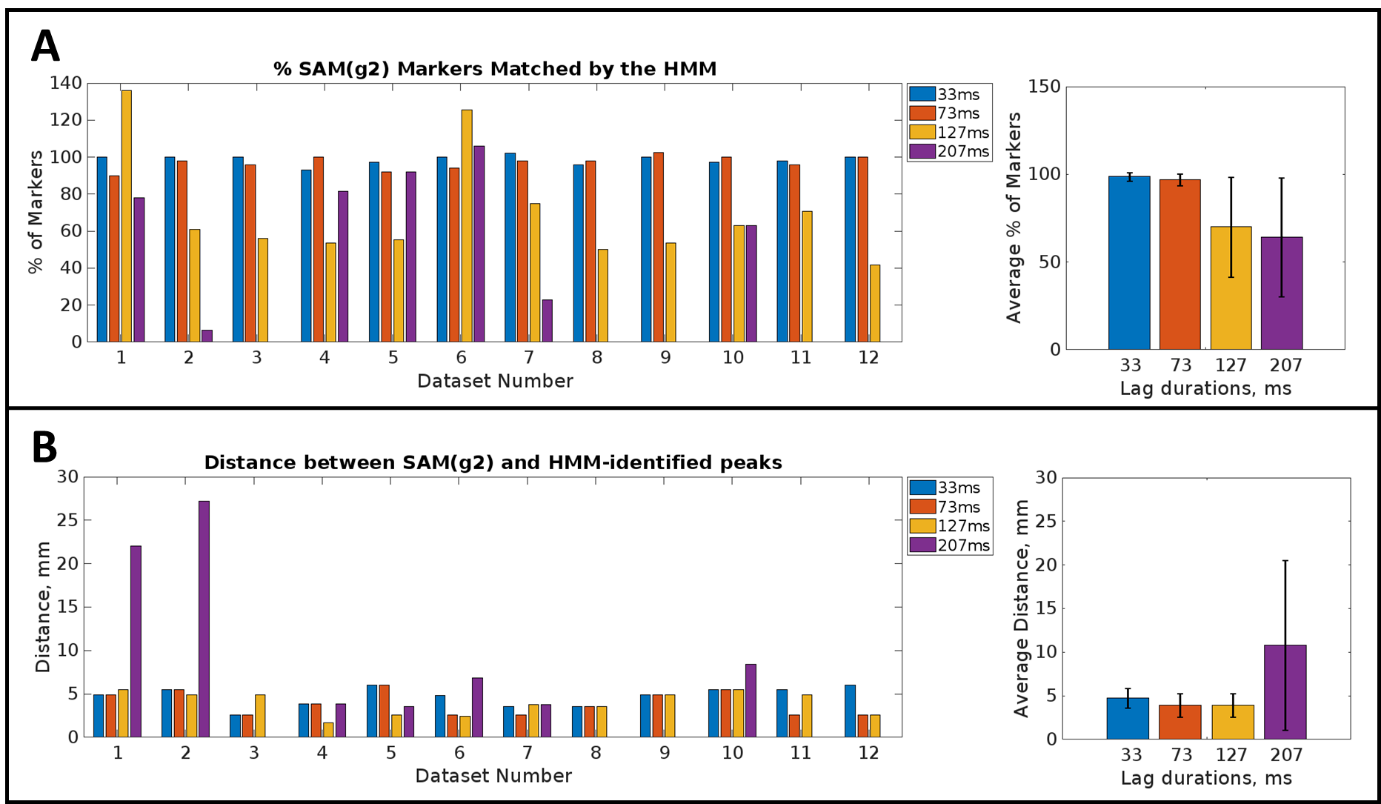
Figure S2:*** *Varying the lag durations used for model inference changes the result of the HMM temporal and spatial localisations for the epileptiform state in patient 1. Panel A shows the temporal match of the HMM epileptiform state visits with the EKM markers for each two-minute dataset (left) and for the average over all datasets (right). Panel B shows the spatial match of the HMM epileptiform state and SAM(g2) localisations for all datasets (left) and the average over datasets (right). The error bars show the standard deviation over datasets. A lag duration of 73ms (used in this paper) captures a high percentage of the EKM markers with a relatively small difference in localisation between the two methods (<5mm).*

**Further Results**

Each patient represents a unique case study, and the HMM method produces a model of epileptiform activity specific to each individual. The results for those patients not included in the main body of the paper are given here.

***
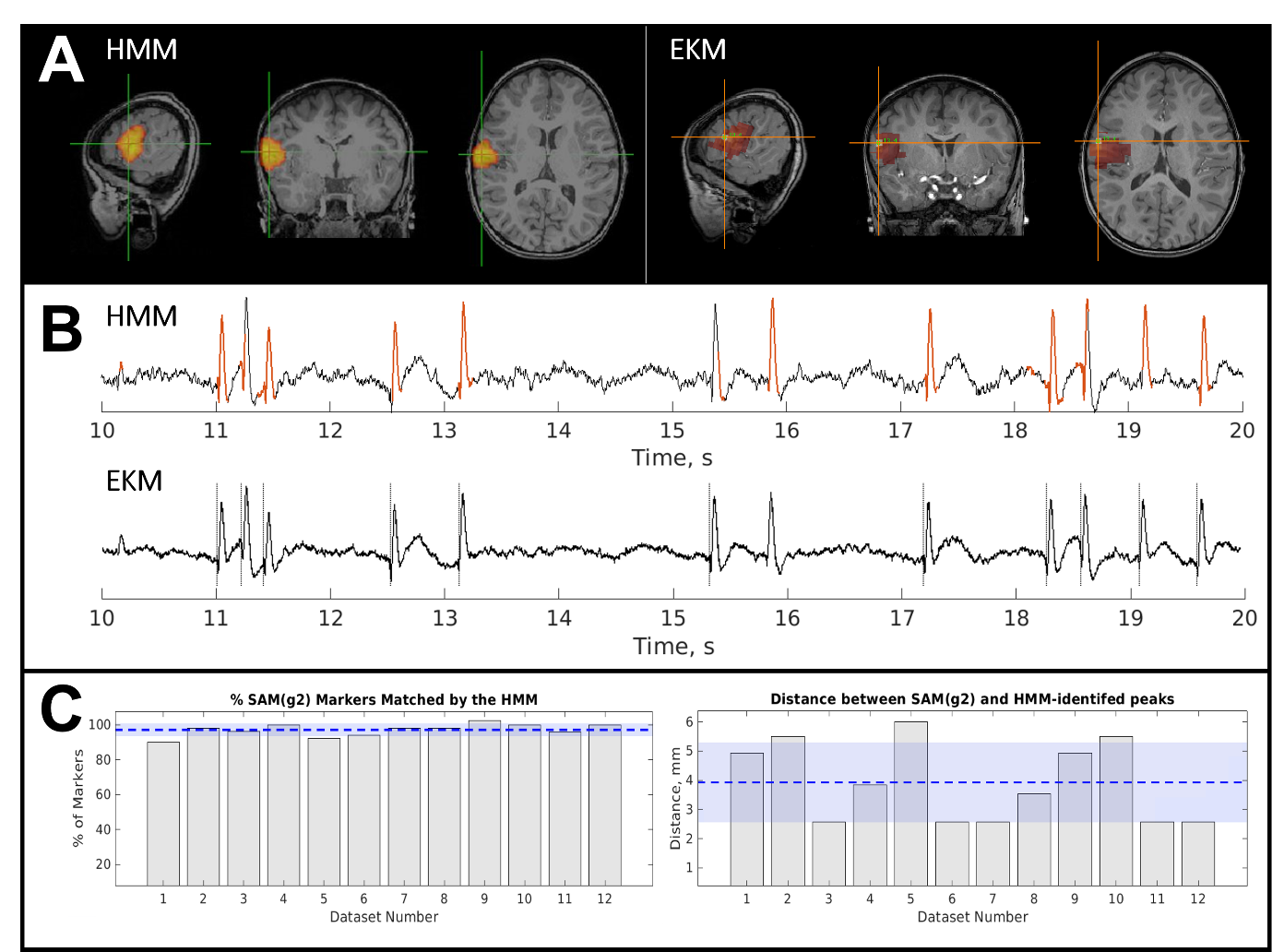
***

***Figure S3: Patient 1 - focal, spike and wave.*** *A) The left-hand side shows the spatial signature of the epileptiform state as defined by the HMM. The right-hand side shows the spatial profile of EKM. Both maps were thresholded for visualization. The time course of neural activity from the peak voxel is shown in B with the HMM state activations shown in red and the EKM markers as dotted lines. The spatial and temporal correspondence between the techniques are quantified in panel C with 4 ± 1mm between peak locations and 97 ± 4% of EKM markers matched by a HMM state visit (mean and standard deviation over 12 2-minute runs). The average length of time spent in the epilepsy state was just 5.4 ± 0.8% (average and SD over runs) of the total time. Note that more than one state visit to a single kurtosis marker will occasionally yield values >100%.*

*
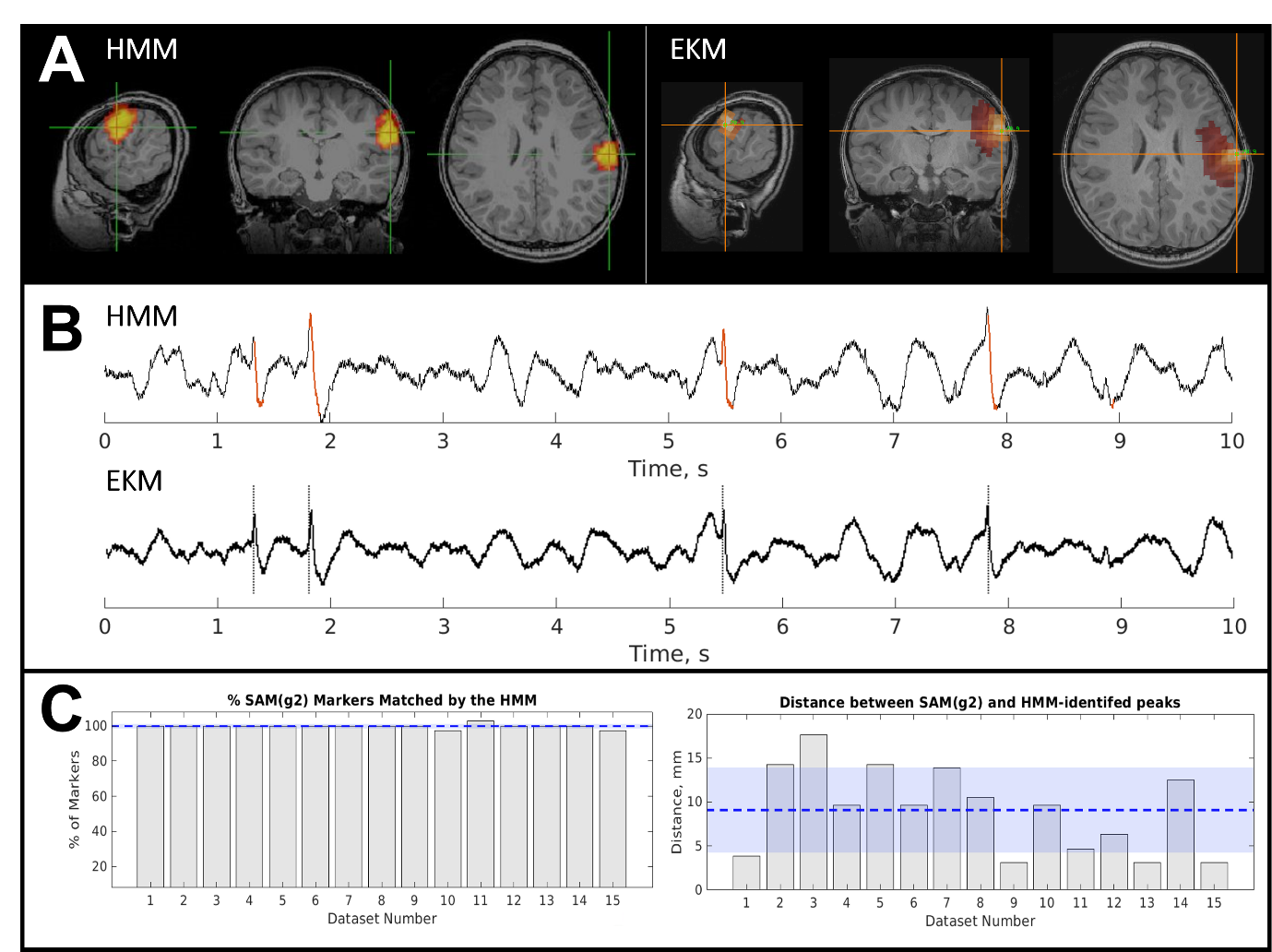
****Figure S4: Patient 3 - focal, spike and wave.*** *Panel A shows spatial maps highlighting the location of epileptogenic cortex using the HMM (left) and EKM (right). The time courses of activity from the peak locations are shown in B for the HMM (upper) and EKM (lower). The HMM state visits are highlighted in red and the EKM markers are shown as black dotted lines. There is good spatial and temporal agreement between techniques (C) with 100 ± 1% of kurtosis markers being matched by an HMM state visit and 9 ± 5mm between peak locations (average and SD over runs). For this patient, the epilepsy state was active for 3.3 ± 0.5% of the total time.*

*
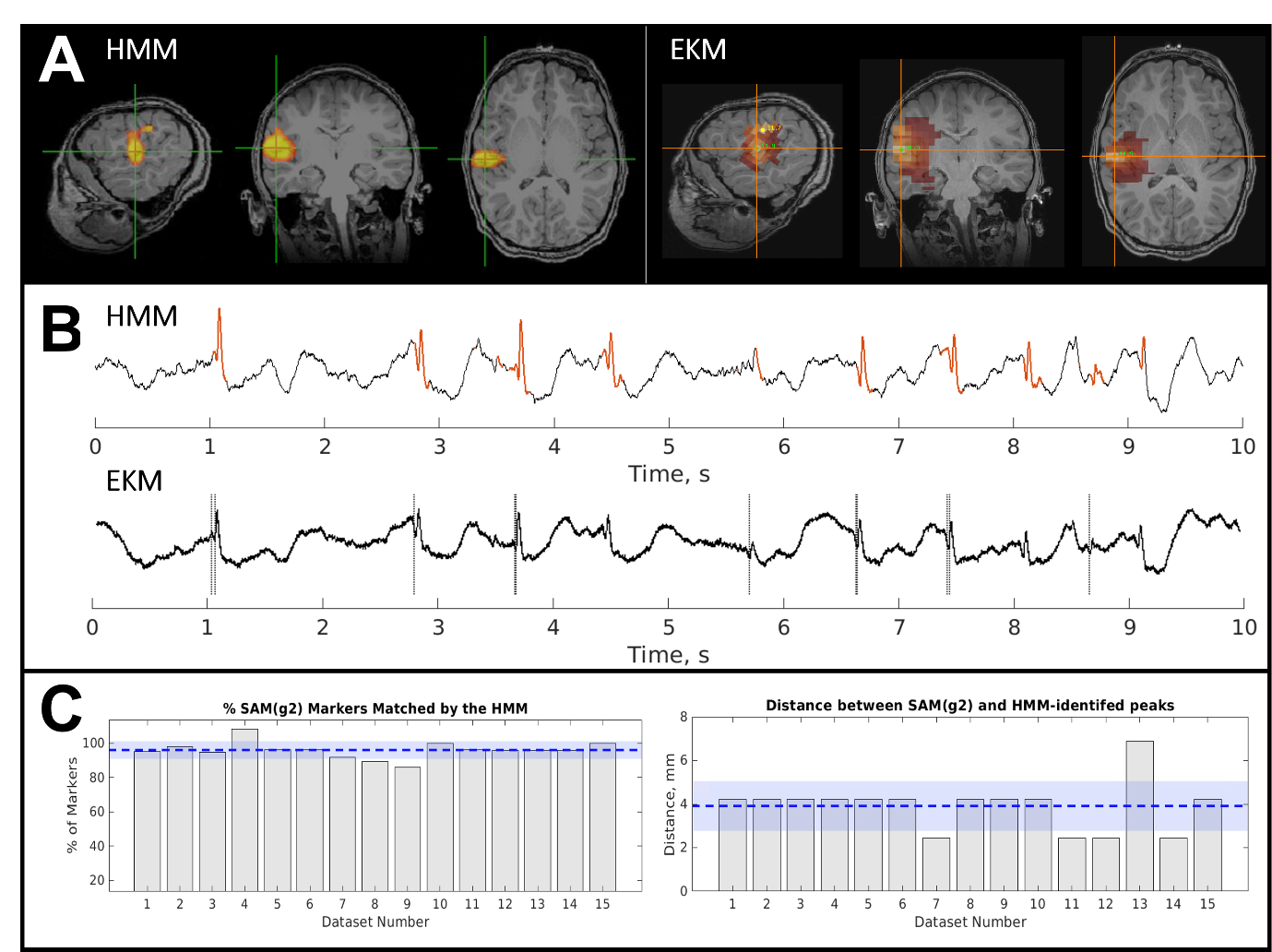
****Figure S5: Patient 4 - focal, spike and wave.*** *There is good spatial agreement between the two methods for this patient as shown in panel A (qualitatively) and quantitatively in panel C (right). There was on average 3.9 ± 0.3mm between the peak locations. The time course of activity from the peak locations for both techniques is shown in panel B. HMM state visits are highlighted in red, and the dotted lines are temporal markers from the EKM method. There is also an excellent temporal match between the two methods with 96 ± 5% of kurtosis markers matched by an HMM state visit (C, left). The epilepsy state was active for 10.4 ± 0.4% of the total time.*


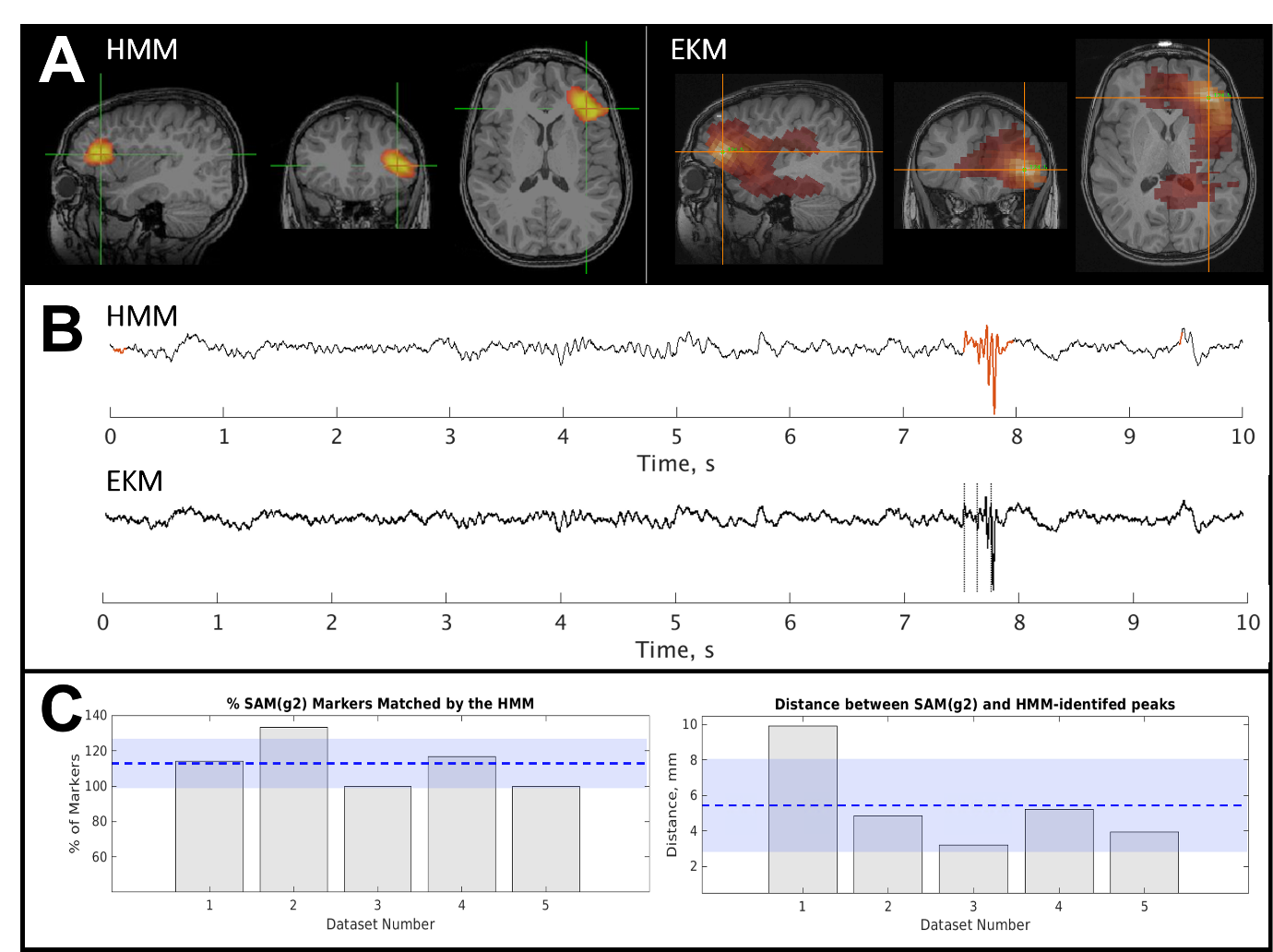


***Figure S6: Patient 6 - focal, polymorphic.*** *Interictal activity arising from the left frontal lobe was identified by both the HMM and EKM methods (A). The virtual depth electrode time course extracted from the peak voxel for both methods is shown in B with HMM state visits highlighted in red and kurtosis markers shown as dotted lines. On average over the five runs, 113 ± 14% of kurtosis markers were matched by an HMM state visit. Note that values greater than 100 are caused by multiple HMM state visits matching with a single kurtosis marker. The distance between the spatial localization for the two methods was 5 ± 3mm. The HMM state was active for 1.1 ± 0.4% of the total time.*

***Figure S7: Patient 7 - multifocal, polymorphic.*** *There was a single HMM epilepsy state associated with each dataset (run) which localized to either the right temporal lobe (Ai) or the left frontal area (Aii). Time courses of activity from the peak of those spatial maps is shown in B with the temporal lobe exhibiting some sharp activity (Bi) and the frontal areas exhibiting spikes (Bii). HMM state visits are highlighted in red. The EKM method identified both the right temporal lobe and left frontal lobe activity in datasets where both were present but the HMM did not. In the two datasets with left frontal activity the HMM failed to identify the temporal lobe activity*
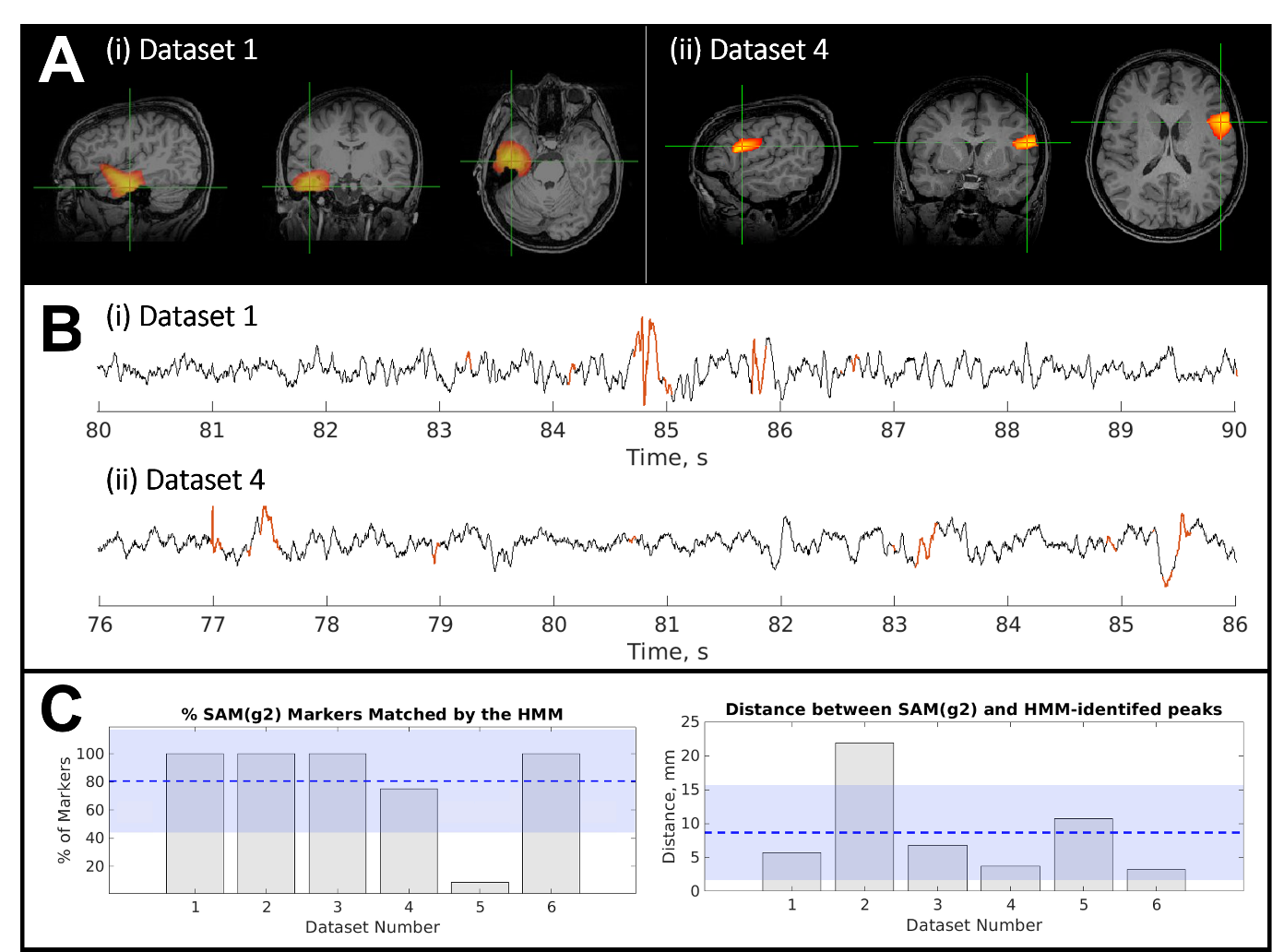
*(so is outperformed by the EKM). This is likely to be because there was a heartbeat-related state which localized to the same location, possibly occluding the epileptiform signal in this case. Using ICA to remove the heartbeat from the data prior to analyses may remedy this in the future. However, for those peak locations that were identified by the HMM, the Euclidian difference between them and the corresponding EKM peak location was 9 ± 7mm (average and SD over runs). The number of EKM markers matched by an HMM state visit was 81 ± 37%, with the HMM state active for 5 ± 5% of the total time. So there was still good agreement between methods.*

Patient 9 was another multi-focal epilepsy patient with a previous tissue resection in the frontal lobe. An EKM analysis showed two locations with elevated kurtosis, one close to the previous left frontal resection, and the second in right parietal cortex. Initially, the parietal activity had been dismissed as sharply contoured mu rhythm. However, the HMM showed that, in 6/13 datasets, a single HMM state described the activity in both brain locations. In other words, the state variance was high in both locations at the same time, implying that the activity (whilst of unclear clinical significance) is, in fact related. This is the case for the data shown in Figure S8A, which depicts the two separate locations in a single map and associated time courses from the two peaks. However, in a further 6/13 datasets the activity was parsed into two states (shown for dataset 2 in B as an orange and blue state). In the remaining dataset there was only the activity from the parietal location, with no apparent activity close to the resection zone.


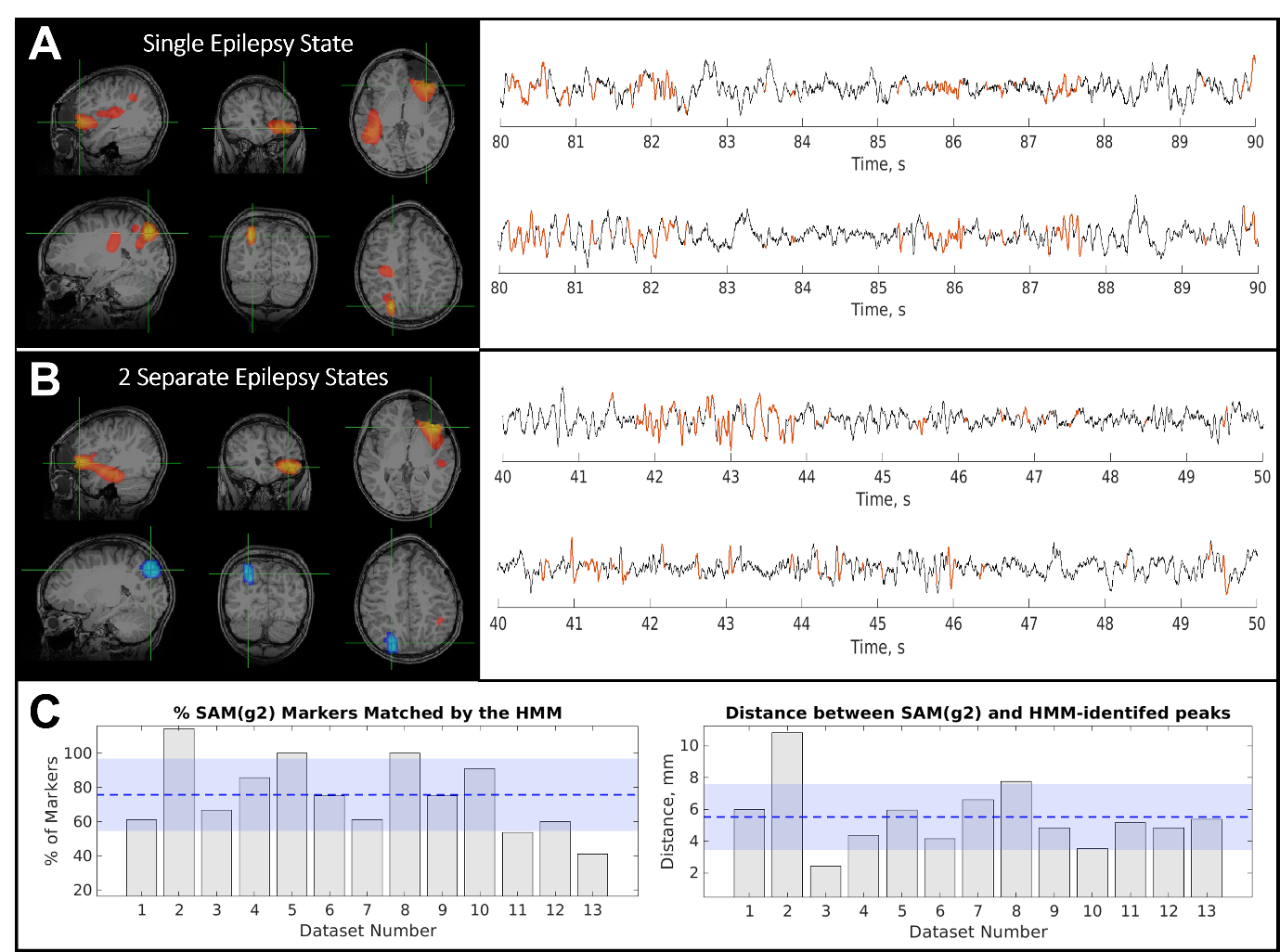


***Figure S8: Patient 9 - multi-focal, polymorphic bursts.*** *A) a single run in which multiple brain locations, determined to be generating epileptiform activity, have been identified in the same state. B) a different run in the same patient where the HMM has split the candidate brain regions across states. Spatial maps were thresholded for visualization. C) the temporal (left) and spatial (right) match between the HMM and EKM. 76 ± 21% of EKM markers were matched by an HMM state visit and the average Euclidian distance between HMM peaks and the corresponding EKM peak was 6 ± 2mm. The total time spent in an epilepsy state was 16 ± 6% of the total time.*
